# Intratumoral Hypoxia Triggers Mitochondrial BHLHE40 ROS Sensing Pathway to Promote Radioresistance in Triple‐Negative Breast Cancer

**DOI:** 10.1002/advs.76864

**Published:** 2026-07-27

**Authors:** Jia Liu, Ziliang Nie, Xi Chen, Guangyu Ji, Yajing Zhang, Zhiqun Zhao, Yuhong Zhang, Xinlong Du, Zhenzhen Zhou, Jiayi Li, Yaozong Yang, Fengqi Sun, Zhibo Yan, Haiquan Lu

**Affiliations:** ^1^ Advanced Medical Research Institute Cheeloo College of Medicine Shandong University Jinan Shandong China; ^2^ Department of General Surgery Qilu Hospital Cheeloo College of Medicine Shandong University Jinan Shandong China; ^3^ School of Basic Medical Sciences Cheeloo College of Medicine Shandong University Jinan Shandong China; ^4^ Key Laboratory for Experimental Teratology of the Ministry of Education Cheeloo College of Medicine Shandong University Jinan Shandong China; ^5^ State Key Laboratory of Discovery and Utilization of Functional Components in Traditional Chinese Medicine Shandong University Jinan Shandong China

**Keywords:** BHLHE40, intratumoral hypoxia, mitochondria, radioresistance, reactive oxygen species (ROS), triple‐negative breast cancer (TNBC)

## Abstract

Intratumoral hypoxia is a hallmark of triple‐negative breast cancer (TNBC) and induces complex biological responses, including treatment resistance and mitochondrial production of reactive oxygen species (ROS). However, the direct mechanisms through which hypoxia‐induced ROS are sensed and contribute to therapeutic resistance remain elusive. ROS can regulate the function of transcription factors through oxidation of cysteine thiol groups, but the compartmentalization limits their physical interaction with transcription factors. Here, BHLHE40, a transcription factor traditionally recognized for its nuclear function, is identified as a novel mitochondrial sensor of hypoxia‐induced ROS. Mitochondrial BHLHE40 experiences ROS‐dependent oxidation of cysteine thiol groups and forms disulfide‐linked homodimers. In addition to post‐translational modification that regulates BHLHE40 protein levels, hypoxia also increases BHLHE40 mRNA levels through hypoxia‐inducible factors (HIFs)‐dependent transcriptional activation. These dual mechanisms of modulating BHLHE40 ensure its rapid elevation during the early stage of hypoxia. Functionally, BHLHE40 plays a critical role in hypoxia‐induced radioresistance through transcriptional activation of cellular antioxidant systems and inhibition of cytotoxic effects mediated by irradiation‐generated ROS. This study reveals a previously unrecognized role of BHLHE40 in sensing and regulating ROS in response to hypoxia, and highlights its potential as a therapeutic target to overcome hypoxia‐promoted radioresistance in TNBC.

## Graphical abstract

Hypoxia triggers a dual mechanism for BHLHE40 upregulation: reactive oxygen species (ROS)‐dependent post‐translational modification and hypoxia‐inducible factor (HIF)‐dependent transcriptional activation. Under hypoxic conditions, mitochondrial BHLHE40 accumulates and senses ROS via cysteine thiol oxidation and disulfide‐linked homodimer formation, while transcriptionally activating antioxidant enzymes and SLC7A11 to suppress intracellular ROS, thereby promoting hypoxia‐induced radioresistance in triple‐negative breast cancer.

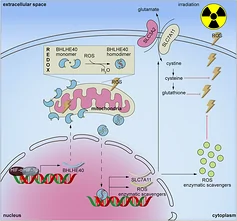

## 1 Introduction

Triple‐negative breast cancer (TNBC), which is characterized by the absence of estrogen receptor (ER), progesterone receptor (PR), and human epidermal growth factor receptor 2 (HER2), remains one of the most significant therapeutic challenges due to its aggressive behavior, lack of effective targeted treatments, and poor prognosis [1]. A hallmark of TNBC is intratumoral hypoxia, which occurs as a result of the imbalance between the high oxygen demand of rapidly proliferating cancer cells and the limited oxygen supply from the disorganized and dysfunctional microvasculature [2]. The hypoxic microenvironment induces a series of adaptive biological responses in TNBC cells that promote tumor progression, therapy resistance, and poor patient outcomes [2, 3, 4]. Considering the ubiquity and biological significance of hypoxia in TNBC [5], understanding the effects and underlying mechanisms of hypoxia on cancer cells is critical for improving treatment strategies for patients with TNBC.

Hypoxia induces complex biological responses, including mitochondrial production of reactive oxygen species (ROS), such as superoxide (O_2_^−^) and hydrogen peroxide (H_2_O_2_) [2, 6, 7]. As an important signaling molecule in redox‐dependent signal transduction, ROS can regulate the function of proteins through post‐translational modifications (PTMs), such as the oxidation of cysteine thiol groups and the formation of disulfide bonds [6, 7, 8]. These oxidative changes can alter the activity of redox‐sensitive transcription factors, thereby affecting gene expression programs and cellular behavior under hypoxic stress [9]. However, a paradox is raised by ROS compartmentalization that the majority of hypoxia‐induced ROS, which are generated in mitochondria, have less opportunity to directly oxidize cysteine thiol groups of nuclear transcription factors, especially considering the presence of powerful ROS‐eliminating enzymes. Although extensive research has highlighted the role of ROS in cancer biology, the direct mechanisms through which hypoxia‐induced ROS influence transcriptional regulation in TNBC remain elusive.

Hypoxia‐inducible factors (HIFs) are master regulators of the cellular response to oxygen deprivation and play a central role in cancer cell adaption to hypoxic environments [10]. HIFs are heterodimers composed of a constitutively expressed β subunit (HIF‐β) and an oxygen‐sensitive α subunit (HIF‐α) [10, 11]. Under normoxic conditions, HIF‐α is rapidly degraded via the ubiquitin‐proteasome pathway through hydroxylation by prolyl hydroxylases (PHDs). However, under hypoxic conditions, the inhibition of PHD activity prevents HIF‐α degradation, allowing it to dimerize with HIF‐β and activate the transcription of HIF‐targeted genes [10, 11, 12, 13]. Activated HIFs drive the transcription of genes involved in angiogenesis, metabolism, survival, invasion, and metastasis [10]. In TNBC, HIFs play a critical role in shaping a tumor microenvironment that supports therapeutic resistance, particularly through metabolic reprogramming toward glycolysis, which decreases mitochondrial function and ROS production [14, 15]. Reduction in ROS accumulation diminishes the effectiveness of therapies that rely on ROS‐induced cell death, such as radiotherapy [16].

Radiotherapy is a widely used therapeutic approach for TNBC patients and has been shown to reduce local recurrence and improve survival in specific patient populations [17, 18]. A meta‐analysis demonstrated that radiotherapy following breast‐conserving surgery significantly reduces the 10‐year recurrence rate and the 15‐year cancer‐related mortality in TNBC patients [19]. However, a recent phase III randomized trial has reported that post‐mastectomy radiotherapy provides improvement only in selected TNBC patients [20]. Extensive studies have revealed that the efficacy of radiotherapy is limited by hypoxia [21, 22, 23]. Hypoxia reduces oxidative damage caused by radiation‐induced free radicals, thereby impairing DNA damage and diminishing the cytotoxic effects of radiotherapy [2, 24]. Therefore, targeting hypoxia may serve as a promising therapeutic strategy to overcome radiotherapy resistance in TNBC.

In this study, we identify BHLHE40, a transcription factor traditionally recognized for its nuclear function, as a direct sensor of hypoxia‐induced ROS. We demonstrate that, in addition to its nuclear localization, BHLHE40 also accumulates in the mitochondrial matrix, where it senses hypoxia‐induced ROS through the formation of a disulfide‐linked homodimer. To ensure rapid cellular response during the early stage of hypoxia, a dual‐mechanism of BHLHE40 upregulation is involved: ROS‐dependent post‐translational stabilization and HIF‐dependent transcriptional activation. Functionally, we show that BHLHE40 promotes hypoxia‐induced radiotherapy resistance by decreasing ROS levels through the transcriptional upregulation of a broad network of enzymatic ROS scavengers.

## 2 Results

### 2.1 Establishment of a TNBC‐Specific Hypoxia‐Prognosis Model

To systematically evaluate changes of gene expression under hypoxic conditions and explore therapeutic targets in hypoxic TNBC, we developed a TNBC‐specific hypoxia‐related gene expression profile by integrating RNA sequencing (RNA‐seq) data and clinical information from TNBC patients in the TCGA_BRCA dataset with 200 hypoxia‐upregulated genes from the Molecular Signatures Database (MSigDB) (Figure 1a) [25]. We performed univariate Cox regression analysis to explore the potential prognostic value of each gene and identified nine genes significantly associated with hypoxia prognosis (Figure 1b). To avoid the potential risk of overfitting, we further applied the least absolute shrinkage and selection operator (LASSO) regression algorithm and narrowed the list to seven genes at a minimum lambda of 0.01 (Figure 1c), and constructed a hypoxia‐prognosis model in TNBC by calculating hypoxia scores (Figure 1d; Table S1). The hypoxia scores from our hypoxia‐prognosis model were strongly correlated with those from another eight independent hypoxia signatures (Figure 1e) [26]. The robust performance of the hypoxia‐prognosis model was validated in an independent dataset of 107 TNBC patients (GSE103091) [27, 28] with area under the curve (AUC) values greater than 0.7 (Figure 1f). Kaplan‐Meier analysis revealed that high hypoxia scores were associated with decreased overall survival in TNBC patients from the TCGA_BRCA and GSE103091 datasets (Figure 1g).

**FIGURE 1:**
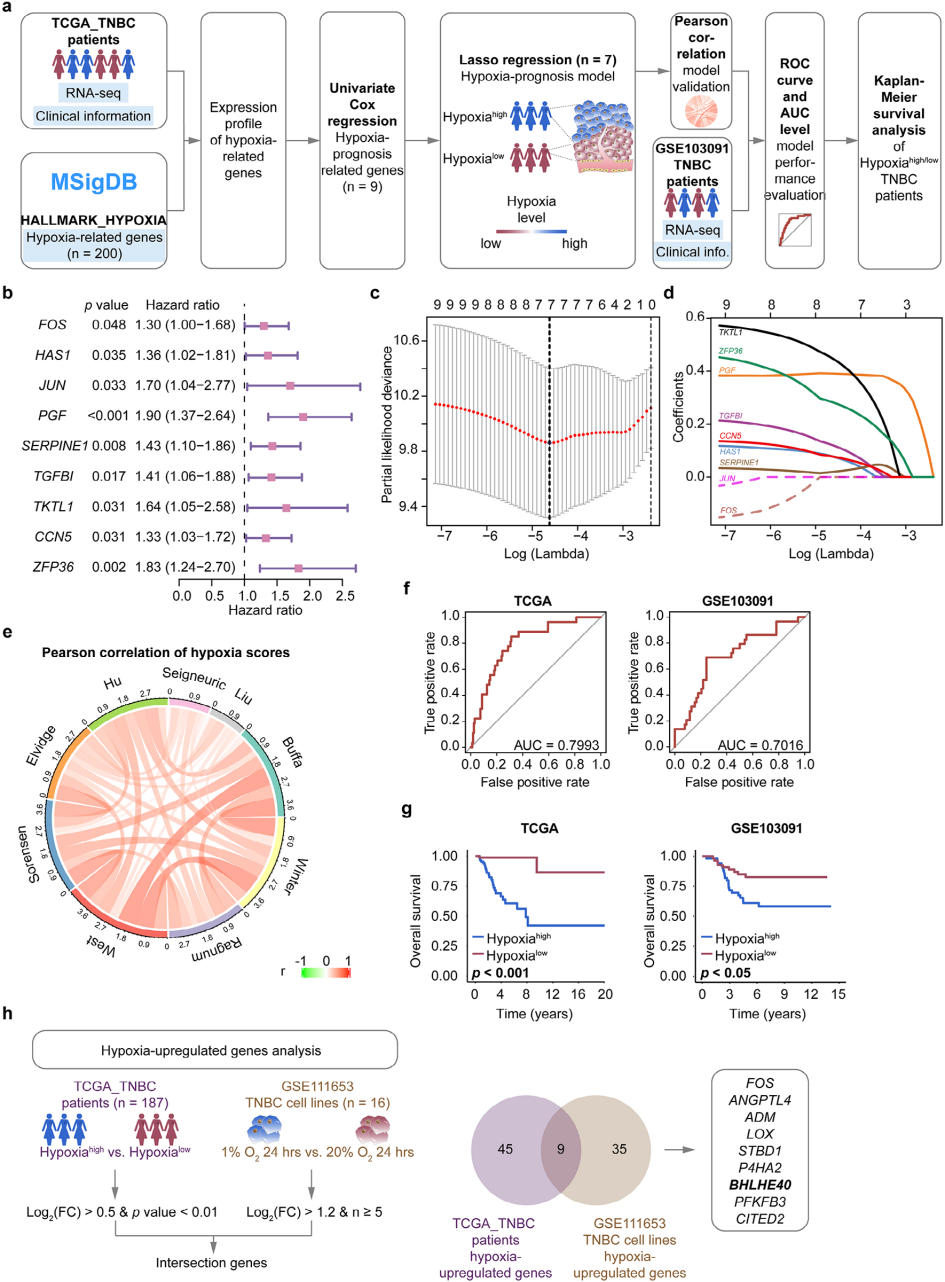
Establishment of a TNBC‐specific hypoxia‐prognosis model. (a) Flowchart of establishment and validation of the TNBC‐specific hypoxia‐prognosis model. (b) Univariate Cox regression of 200 hypoxia‐upregulated genes downloaded from MSigDB in the TCGA_TNBC cohort. Left column, nine hypoxia‐prognosis related genes that have a *p‐*value <0.05. Pink cube, hazard ratio. Purple line segment, 95% confidence interval of hazard ratio. (c) The adjustment penalty parameter Lambda was selected in the LASSO regression by cross validation. Upper *x*‐axis, the number of genes. Lower *x*‐axis, Log (Lambda). *Y*‐axis, partial likelihood deviance. Grey line segment, 95% CI of partial likelihood deviance. Left dash line, minimum Lambda. (d) LASSO regression of nine hypoxia‐prognosis‐related genes in the TCGA_TNBC cohort. Upper *x*‐axis, the number of genes. Lower *x*‐axis, Log (Lambda) *y*‐axis, coefficients. Color curves, nine hypoxia‐prognosis‐related genes. (e) Pearson correlation of hypoxia scores from our hypoxia‐prognosis model with eight independent hypoxia signatures. The thickness and color of the ribbons is associated with the correlation of hypoxia scores in the TCGA_TNBC cohort. (f) Receiver operating characteristic (ROC) analysis of our hypoxia‐prognosis model in the TCGA_TNBC (left) and GSE103091 (right) cohorts. AUC, area under the curve. (g) Kaplan–Meier analysis of the overall survival of patients from TCGA_TNBC (left) and GSE103091 (right) stratified according to our hypoxia‐prognosis model. (h) Flowchart of screening process in primary TNBC tumor samples and cell lines. n = 187, the number of TCGA_TNBC patients. n = 16 / n ≥5, the number of TNBC cell lines. hrs, hours. FC, fold change; *p* values were determined by using univariate Cox regression (b), Pearson correlation analysis (e), and log‐rank tests (g).

Using the median value of the hypoxia score, we stratified TNBC patients from TCGA_BRCA into hypoxia^high^ and hypoxia^low^ groups. We compared gene expression profiles between the two groups and identified 54 upregulated genes in hypoxia^high^ group (Table S2). We also selected TNBC samples from GSE111653 dataset, which contains RNA‐seq data of 32 breast cancer cells or normal human mammary epithelial cells exposed to 20% O_2_ or 1% of O_2_ for 24 h [29, 30], compared RNA expression data, and identified 44 upregulated genes in hypoxia‐exposed TNBC cells (Table S3). We identified nine common hypoxia‐upregulated genes (*FOS*, *ANGPTL4*, *ADM*, *LOX*, *STBD1*, *P4HA2*, *BHLHE40*, *PFKFB3*, *CITED2*) in both primary TNBC tumor samples and cell lines (Figure 1h).

### 2.2 BHLHE40 is Present in the Mitochondria, and Hypoxia Induces Mitochondrial BHLHE40 Protein Levels

Among the nine common hypoxia‐upregulated genes, three (*FOS*, *BHLHE40*, and *CITED2*) are transcription factors that play their function in the cell nucleus [31, 32, 33]. Unexpectedly, when we explored BHLHE40 subcellular localization in the TNBC cell line MDA‐MB‐468 by immunoelectron microscopy, we discovered the presence of BHLHE40 in the mitochondria, and the mitochondrial BHLHE40 levels were induced when the cells were exposed to 1% of O_2_ for 2 or 24 h (Figure 2a). To validate this observation, we generated shRNA‐mediated scramble or BHLHE40 knockdown subclones in another TNBC cell line MDA‐MB‐231 (Figure S1a,b), and examined BHLHE40 subcellular localization in response to hypoxia. Although the majority of BHLHE40 was located in the nucleus (Figure 2c), we discovered the presence of BHLHE40 in the mitochondria in scrambled control but not BHLHE40 knockdown subclones of MDA‐MB‐231 cells (Figure 2b), confirming the specificity of the antibody we used for immunoelectron microscopy. We also found that exposure of MDA‐MB‐231 cells to hypoxia for 2 or 24 h significantly induced mitochondrial BHLHE40 levels (Figure 2b). In addition, we performed immunofluorescence staining in MDA‐MB‐231 cells and demonstrated that a subset of BHLHE40 was co‐localized with mitochondria marker TOM20, and the proportion of BHLHE40 and TOM20‐labeled mitochondria co‐localization was increased when the cells were exposed to 1% of O_2_ for 2 or 24 h (Figure 2d). Taken together, these data demonstrate that BHLHE40 is present in the mitochondria and hypoxia induces mitochondrial BHLHE40 protein levels.

**FIGURE 2:**
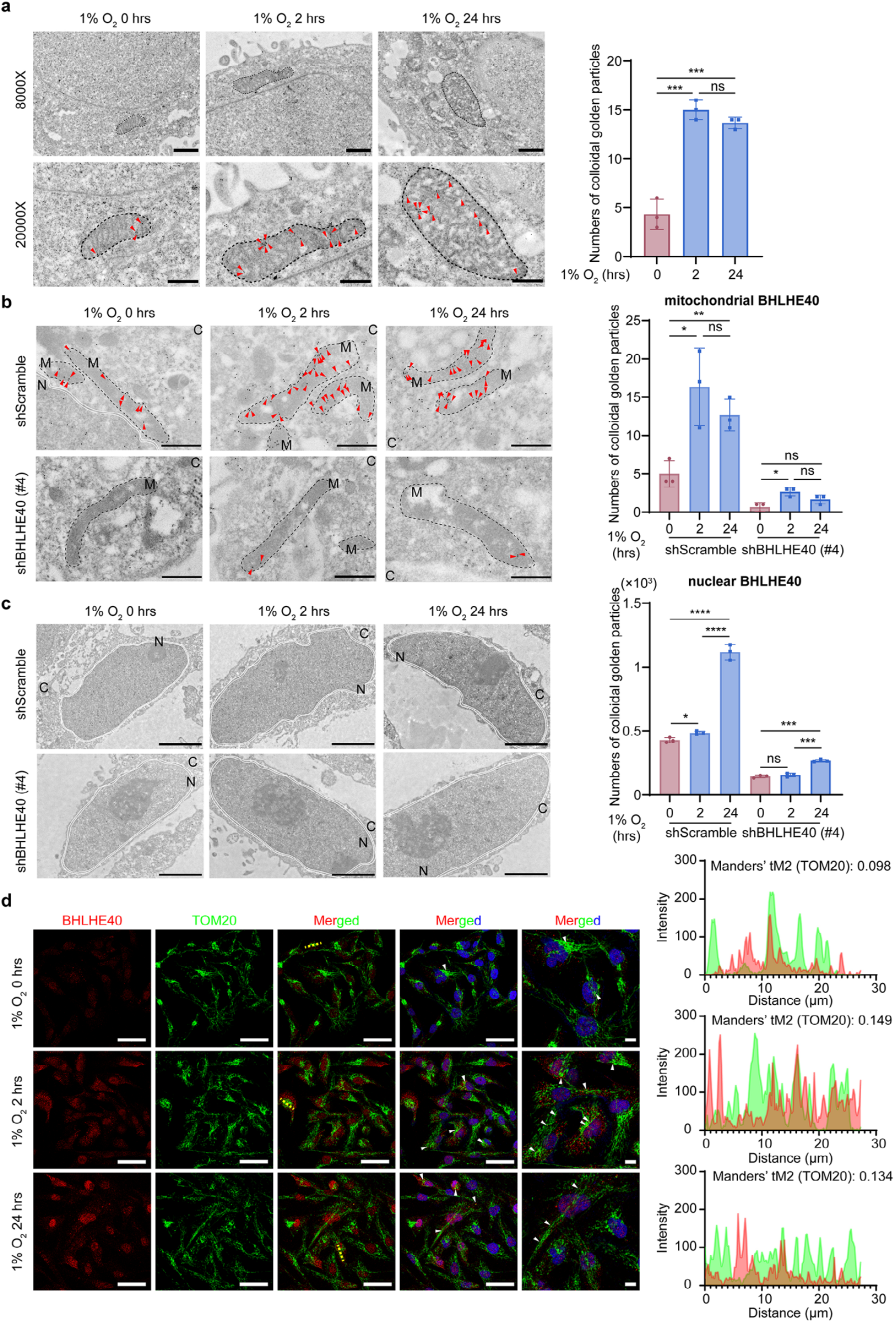
BHLHE40 is present in the mitochondria, and hypoxia induces mitochondrial BHLHE40 protein levels. (a) Representative immunoelectron microscopy images (left) and numbers of colloidal golden particles (right) of MDA‐MB‐468 cells under 1% of O_2_ for 0, 2, or 24 h. Area enclosed by dashed lines, representative mitochondria. Red arrows and black colloidal golden particles, BHLHE40 proteins. Scale bars, 1 µm (8000X) and 500 nm (20000X). (b) Representative immunoelectron microscopy images (left) and numbers of colloidal golden particles (right) of scrambled control and BHLHE40 knockdown subclones of MDA‐MB‐231 cells under 1% of O_2_ for 0, 2, or 24 h. Area enclosed by black dashed lines, representative mitochondria. Area enclosed by double white solid lines, representative nucleus. Red arrows and black colloidal golden particles, BHLHE40 proteins. C, cytoplasm. M, mitochondria. N, nucleus. Scale bars, 500 nm. (c) Representative immunoelectron microscopy images (left) and numbers of colloidal golden particles (right) of scrambled control and BHLHE40 knockdown subclones of MDA‐MB‐231 cells under 1% of O_2_ for 0, 2, or 24 h. Area enclosed by double white solid lines, representative nuclear. Black colloidal golden particles, BHLHE40 proteins. C, cytoplasm. N, nucleus. Scale bars, 2 µm. #4, different BHLHE40 shRNA sequence. (d) Immunofluorescence staining images of BHLHE40 and TOM20 in MDA‐MB‐231 cells under 1% of O_2_ for 0, 2, or 24 h (left) and intensity profiles in each channel along highlighted yellow dashed lines in the merged images (right). Red, BHLHE40. Green, TOM20. Blue, DAPI. White arrows, BHLHE40 and TOM20 co‐localization. Scale bars, 50 and 10 µm. Manders’ tM2 (TOM20), the proportion of the signal intensity in BHLHE40 channel that co‐localize with TOM20 channel relative to the total signal intensity in TOM20 channel hrs, hours. *p* values were determined by using one‐way ANOVA (a, b, c; n = 3, mean ± SD). ^*^, *p* < 0.05; ^**^, *p* < 0.01; ^***^, *p* <0.001; ^****^, *p* <0.0001; ns, not significant.

### 2.3 BHLHE40 Functions as a Sensor of Hypoxia‐induced ROS Through Formation of Disulfide‐linked Homodimer

Next, we investigated the submitochondrial localization of BHLHE40 by mitochondrial protease protection assay in MDA‐MB‐231 and MDA‐MB‐468 cells. BHLHE40 was protected from Proteinase K degradation in intact mitochondria, whereas the outer membrane control TOM20 was completely digested; upon membrane permeabilization with Triton X‐100, BHLHE40 became accessible to degradation. These results demonstrate that BHLHE40 resides in the mitochondrial matrix (Figure 3a). Because mitochondria are the primary source of ROS [2, 6, 7], and BHLHE40 contains multiple cysteine residues that can sense and transduce changes of cellular redox status (Figure S2a,b) [34, 35], we hypothesized that mitochondrial matrix‐localized BHLHE40 may function as a ROS sensor through oxidation of cysteine thiol groups and formation of disulfide‐linked homodimer (Figure S2c). Exposure of MDA‐MB‐231 cells to hypoxia for 2 h induced intracellular ROS levels, which were blocked by pre‐treatment with ROS inhibitor N‐acetyl‐cysteine (NAC) (Figure S2d,e). Therefore, to test our hypothesis, we exposed MDA‐MB‐231 cells to hypoxia for 2 or 24 h, isolated mitochondrial proteins, and performed immunoprecipitation with an antibody against BHLHE40. Immunoprecipitated mitochondrial BHLHE40 demonstrated a dramatic accumulation in response to hypoxia, which was abrogated by pre‐treatment of NAC (Figure 3b), indicating that hypoxia induces mitochondrial BHLHE40 protein levels in a ROS‐dependent manner. We further performed Ellman's assay, a classical method to quantify free thiol groups [36, 37, 38], with immunoprecipitated mitochondrial BHLHE40 proteins. We found that hypoxia significantly increased the levels of total cysteine thiols (Figure 3c) and total free thiols (Figure 3d) in the same amount of mitochondrial BHLHE40 proteins, which were blocked by pre‐treatment of NAC (Figure 3c,d). Most importantly, hypoxia increased the formation of disulfide bonds between cysteine residues in mitochondrial BHLHE40 proteins (calculated by subtracting the amount of free thiols from total cysteine thiols), which were also abrogated by pre‐treatment of NAC (Figure 3e). To directly demonstrate the existence of mitochondrial BHLHE40 homodimer, we compared mitochondrial BHLHE40 levels in non‐reducing and reducing gels, and detected hypoxia‐induced BHLHE40 homodimer in immunoblots under non‐reducing condition in MDA‐MB‐231 and MDA‐MB‐468 cells (red arrows), which completely disappeared under reducing conditions (Figure 3f; Figure S2f).

**FIGURE 3:**
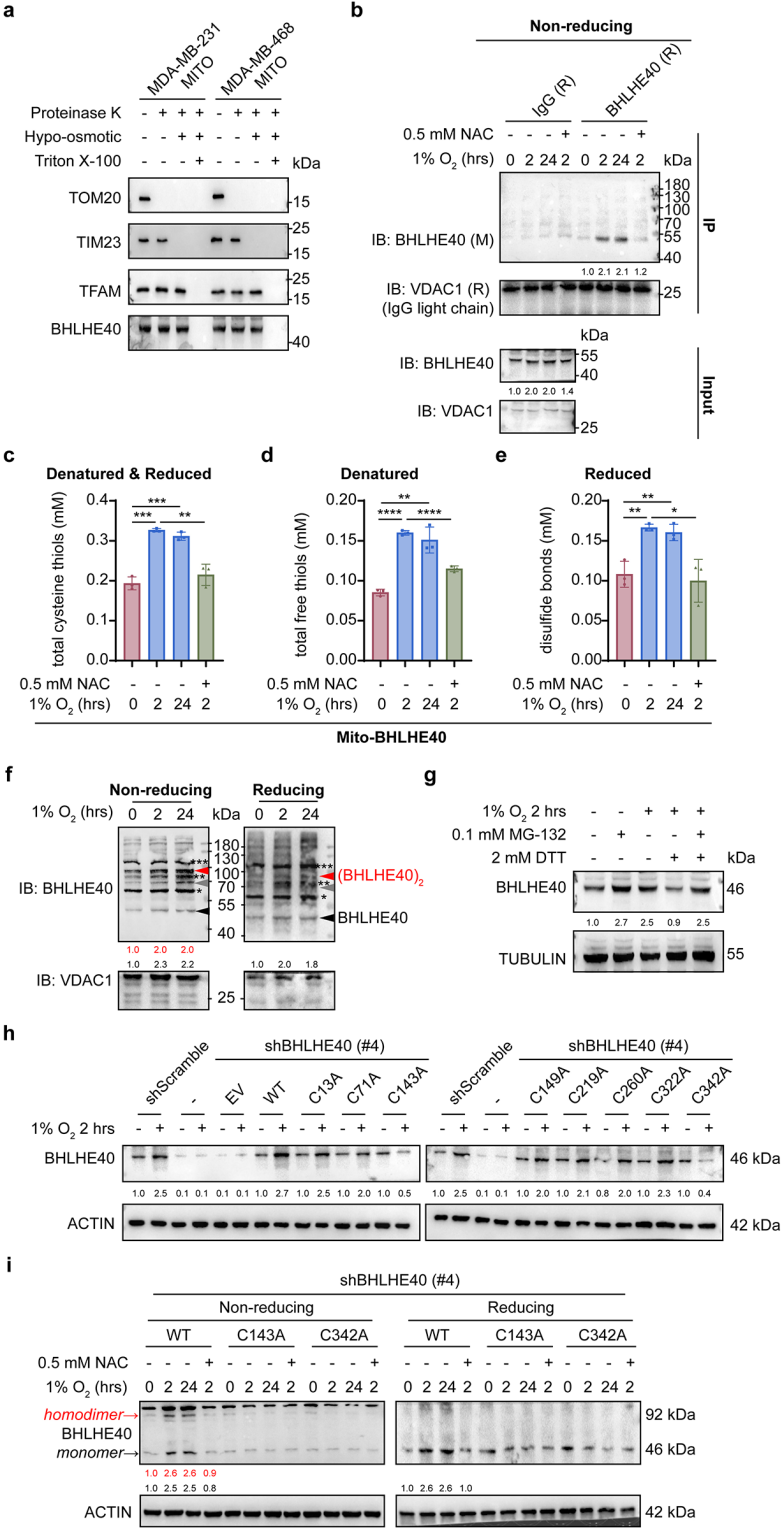
BHLHE40 functions as a sensor of hypoxia‐induced ROS through formation of a disulfide‐linked homodimer. (a) Mitochondrial protease protection assays on isolated mitochondria from MDA‐MB‐231 and MDA‐MB‐468 cells. TOM20, the outer mitochondrial membrane protein. TIM23, the inner mitochondrial membrane protein. TFAM, the mitochondrial matrix protein. (b) Immunoprecipitation of the mitochondrial BHLHE40 proteins in MDA‐MB‐231 cells under 1% of O_2_ for 0, 2, or 24 h with or without 0.5 mm NAC pre‐treatment. R, rabbit. M, mouse. (c) Ellman's assay of both denatured and reduced immunoprecipitated mitochondrial BHLHE40 proteins in MDA‐MB‐231 cells. (d) Ellman's assay of only denatured immunoprecipitated mitochondrial BHLHE40 proteins in MDA‐MB‐231 cells. (e) Ellman's assay of only reduced immunoprecipitated mitochondrial BHLHE40 proteins in MDA‐MB‐231 cells. (f) Non‐reducing and reducing western blot analysis of the mitochondrial BHLHE40 proteins in MDA‐MB‐231 cells under 1% of O_2_ for 0, 2, or 24 h. VDAC1, a mitochondrial protein controls. Red arrows, BHLHE40 homodimers. Grey arrows, BHLHE40 heterodimers formed by BHLHE40 monomers and other proteins via disulfide bonds. Black arrows, BHLHE40 monomers. Asterisks, anti‐BHLHE40 antibody background bands unrelated to disulfide bonds. (g) Western blot analysis of BHLHE40 proteins in MDA‐MB‐231 cells under 1% of O_2_ for 0 or 2 h with or without 0.1 mM MG‐132 and 2 mM DTT pre‐treatment. (h) Western blot analysis of BHLHE40 proteins in scrambled control and BHLHE40 knockdown subclones of MDA‐MB‐231 cells, transfected with EV, WT, or mutant BHLHE40 expression vectors, under 1% of O_2_ for 0 or 2 h. EV, empty vector. WT, wild‐type. C, cysteine. A, alanine. (i) Non‐reducing and reducing western blot analysis of BHLHE40 proteins in BHLHE40 knockdown subclones of MDA‐MB‐231 cells transfected with WT or mutant BHLHE40 expression vectors, under 1% of O_2_ for 0, 2, or 24 h with or without 0.5 mM NAC pre‐treatment. Red arrows, BHLHE40 homodimers. Black arrows, BHLHE40 monomers. NAC, N‐acetyl‐cysteine; hrs, hours. Mito‐BHLHE40, mitochondrial BHLHE40 proteins. *p* values were determined by using one‐way ANOVA (c, d, e; n = 3, mean ± SD). ^*^, *p* < 0.05; ^**^, *p* < 0.01; ^***^, *p* < 0.001; ^****^, *p* < 0.0001.

We found that hypoxia‐induced BHLHE40 was decreased by addition of dithiothreitol (DTT), a strong reducing agent widely used to reduce disulfide bonds, and the effect of DTT was abrogated by proteasome inhibitor MG‐132 (Figure 3g), suggesting that hypoxia‐induced disulfide bonds protect BHLHE40 protein against degradation through the proteasome pathway. To identify the key cysteine residues of BHLHE40 involved in ROS sensing and disulfide bonds formation, we predicted the localization of disulfide bonds in BHLHE40 homodimer protein with AlphaFold 3, and identified an intermolecular disulfide bond between Cys‐143 and Cys‐342, or between Cys‐149 and Cys‐149 (Figure S2g). To experimentally validate these predictions, we generated eight single cysteine‐to‐alanine mutants of BHLHE40 (C13A, C71A, C143A, C149A, C219A, C260A, C322A, and C342A) (Figure S2i), transfected them into BHLHE40‐knockdown MDA‐MB‐231 cells (Figure S2h), and exposed the cells under hypoxia for 2 h. Upon hypoxic exposure, wild‐type (WT) and six of the mutants demonstrated increased BHLHE40 protein levels, while the C143A and C342A mutants did not (Figure 3h), indicating that Cys‐143 and Cys‐342 are essential for BHLHE40 protein stability and hypoxia‐induced accumulation. In addition, only wild‐type BHLHE40, but not C143A and C342A mutants, formed homodimers in response to hypoxia, as shown in the non‐reducing but not reducing gel (Figure 3i), confirming the existence of an intermolecular disulfide bond between Cys‐143 and Cys‐342 of BHLHE40 homodimer. These data demonstrate that mitochondrial BHLHE40 functions as a sensor to hypoxia‐induced ROS through formation of a disulfide‐linked homodimer.

### 2.4 Hypoxia Induces Whole‐Cell BHLHE40 Expression in a ROS‐ and HIFs‐Dependent Manner

Next, we investigated the spatial and temporal expression dynamics of hypoxia‐induced BHLHE40 in TNBC cells. We exposed MDA‐MB‐231 and MDA‐MB‐468 cells to hypoxia for 2 or 24 h, isolated cytoplasmic proteins (Figure 4a; Figure S3a), or mitochondrial and nuclear proteins (Figure 4b), and examined BHLHE40 protein levels in different cellular compartments. We found that hypoxia increased BHLHE40 protein levels in whole‐cell lysates, cytoplasmic, mitochondrial, and nuclear lysates (Figure 4a,b; Figure S3a), suggesting that hypoxia induces a global expression of BHLHE40 in TNBC cells, not limited to mitochondria (Figures 2a–d, 4a,b; Figure S3a). Hypoxia‐induced whole‐cell BHLHE40 protein levels were consistent across several other breast cancer cell lines, including HS578T (TNBC), SUM159 (TNBC), and MCF7 (ER‐positive) (Figure S3b). Hypoxia‐induced whole‐cell BHLHE40 protein levels were abrogated by pre‐treatment with NAC, while exogenous H_2_O_2_ was sufficient to induce whole‐cell BHLHE40 protein levels (Figure 4c; Figure S3c,d), suggesting that hypoxia induces whole‐cell BHLHE40 protein levels in a ROS‐dependent manner. Hypoxia or exogenous H_2_O_2_ only induced protein levels of wild‐type, but not C143A and C342A mutant BHLHE40 (Figure 4d), further demonstrating that Cys‐143‐ and Cys‐342‐mediated disulfide bond formation is required for stabilization and accumulation of BHLHE40 protein levels.

**FIGURE 4:**
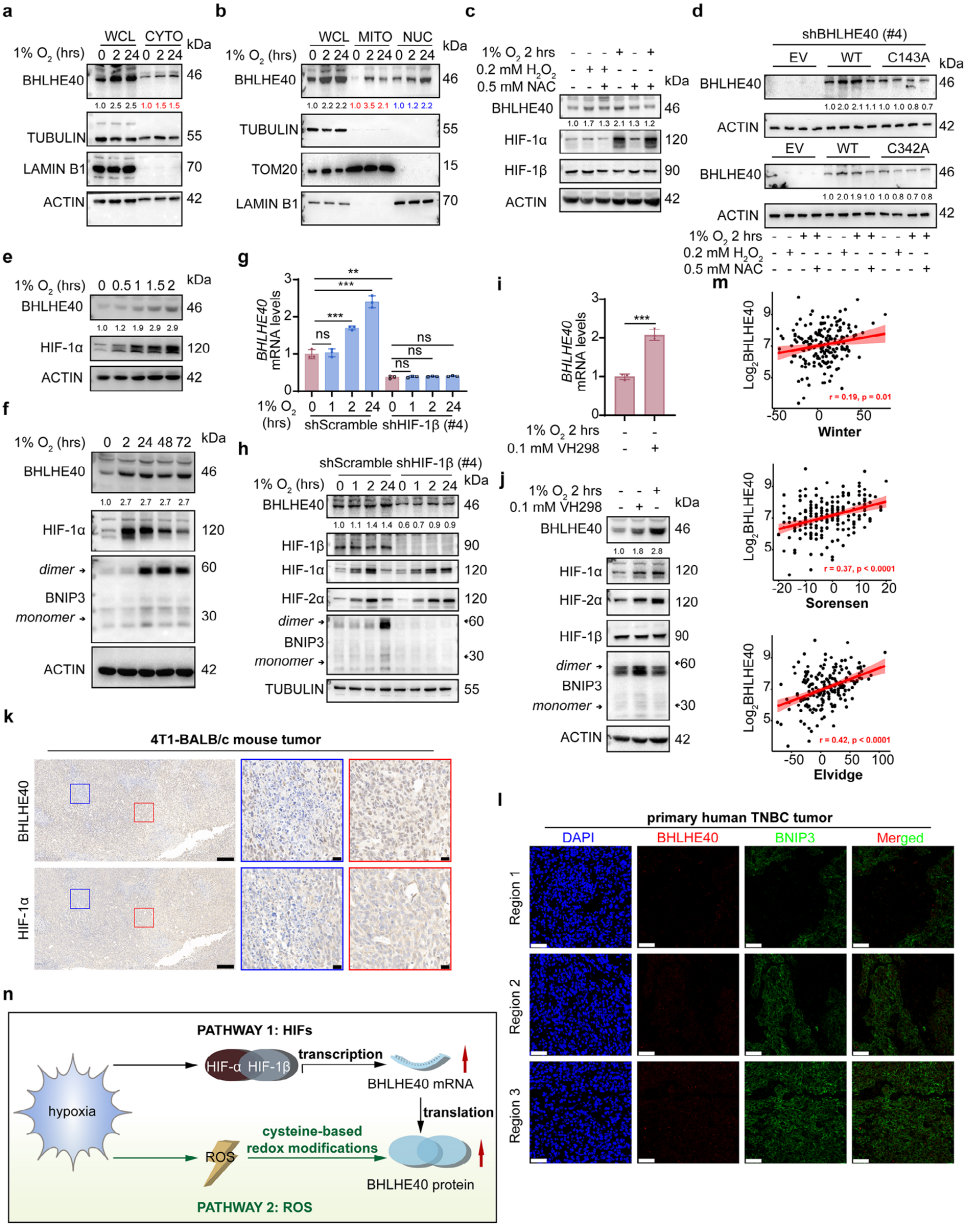
Hypoxia induces whole‐cell BHLHE40 expression in a ROS‐ and HIFs‐dependent manner. (a) Western blot analysis of BHLHE40 proteins in the whole‐cell lysates and cytoplasmic lysates of MDA‐MB‐231 cells under 1% of O_2_ for 0, 2, or 24 h. Tubulin, cytoplasmic protein controls. Lamin B1, nuclear protein control. WCL, whole‐cell lysates. CYTO, cytoplasmic lysates. (b) Western blot analysis of BHLHE40 proteins in the whole‐cell lysates, mitochondrial and nuclear lysates of MDA‐MB‐231 cells under 1% of O_2_ for 0, 2, or 24 h. Tubulin, cytoplasmic protein controls. TOM20, mitochondrial protein controls. Lamin B1, nuclear protein controls. WCL, whole‐cell lysates. MITO, mitochondrial lysates. NUC, nuclear lysates. (c) Western blot analysis of BHLHE40, HIF‐1α and HIF‐1β proteins in MDA‐MB‐231 cells under 1% of O_2_ for 0 or 2 h with or without 0.2 mM H_2_O_2_ and 0.5 mM NAC pre‐treatment. (d) Western blot analysis of BHLHE40 proteins in BHLHE40 knockdown subclones of MDA‐MB‐231 cells transfected with EV, WT, or mutant BHLHE40 expression vectors, under 1% of O_2_ for 0 or 2 h with or without 0.2 mM H_2_O_2_ and 0.5 mM NAC pre‐treatment. EV, empty vector. WT, wild‐type. C, cysteine. A, alanine. NAC, N‐acetyl‐cysteine. (e) Western blot analysis of BHLHE40 and HIF‐1α proteins in MDA‐MB‐231 cells under 1% of O_2_ for 0 to 2 h. (f) Western blot analysis of BHLHE40, HIF‐1α, and BNIP3 proteins in MDA‐MB‐231 cells under 1% of O_2_ for 0, 2, 24, 48, or 72 h. (g) RT‐qPCR analysis of BHLHE40 mRNA levels in scrambled control and HIF‐1β knockdown subclones of MDA‐MB‐231 cells under 1% of O_2_ for 0, 1, 2, or 24 h. (h) Western blot analysis of BHLHE40, HIF‐1β, HIF‐1α, HIF‐2α, and BNIP3 proteins in scrambled control and HIF‐1β knockdown subclones of MDA‐MB‐231 cells under 1% of O_2_ for 0, 1, 2 or 24 h. (i) RT‐qPCR analysis of BHLHE40 mRNA levels in MDA‐MB‐231 cells treated with or without 0.1 mM VH298. (j) Western blot analysis of BHLHE40, HIF‐1α, HIF‐2α, HIF‐1β and BNIP3 proteins in MDA‐MB‐231 cells treated with or without 0.1 mM VH298 under 1% of O_2_ for 0 or 2 h. (k) Immunohistochemical staining images of BHLHE40 and HIF‐1α proteins of serial sections from 4T1‐BALB/c mouse tumor samples. Blue and red boxes, higher magnifications. Scale bars, 200 and 20 µm. (l) Immunofluorescence staining images of BHLHE40 and BNIP3 proteins in different regions from a primary human TNBC tumor. Blue, DAPI. Red, BHLHE40. Green, BNIP3. Scale bars, 50 µm. (m) Pearson correlation analysis of BHLHE40 mRNA levels and mRNA‐based hypoxia signatures from literature (Winter, Sorensen and Elvidge) in the TCGA_TNBC cohort. (n) Graphic illustration of dual mechanisms of whole‐cell BHLHE40 induced by hypoxia hrs, hours. *p* values were determined by using one‐way ANOVA (g; n = 3, mean ± SD), two‐tailed unpaired Student's t‐tests (i; n = 3, mean ± SD) and Pearson correlation analysis (m; n = 187). ^**^, *p* <0.01; ^***^, *p* <0.001; ^****^, *p* <0.0001; ns, not significant.

We also explored the temporal expression pattern of whole‐cell BHLHE40 protein in response to hypoxia in MDA‐MB‐231 and MDA‐MB‐468 cells, and found that BHLHE40 protein was induced within 2 h of hypoxic exposure and remained stable for up to 72 h (Figure 4e,f; Figure S3e,f). This kinetic pattern was distinct from the transient induction of HIF‐1α, which was induced under hypoxia for 2 h but gradually decreased with prolonged hypoxic exposure, and was distinct from the delayed induction of classical HIF‐regulated protein BNIP3 (Figure 4e,f; Figure S3e,f).

To investigate whether HIFs are involved in the regulation of hypoxia‐induced whole‐cell BHLHE40 expression at the early stage of hypoxia, we generated shRNA‐mediated HIF‐1β knockdown subclones in MDA‐MB‐231 and MDA‐MB‐468 cells (Figure S3g). HIF‐1β knockdown suppressed hypoxia‐induced whole‐cell BHLHE40 expression at both mRNA (nearly completely suppressed) (Figure 4g; Figure S3h) and protein (Figure 4h; Figure S3i) levels, suggesting that HIFs are required for hypoxia‐induced whole‐cell BHLHE40 expression. Conversely, stabilization of HIF‐1α and HIF‐2α proteins with VH298, an effective inhibitor of the VHL‐HIFα interaction [39], at a sub‐lethal dose of 0.1 mM (Figure S2d), phenocopied hypoxia‐induced whole‐cell BHLHE40 expression at both mRNA (Figure 4i; Figure S3j) and protein (Figure 4j; Figure S3k) levels.

To investigate changes of whole‐cell BHLHE40 levels in response to hypoxia in vivo, we established a TNBC mouse model by orthotopically injecting 4T1 cells into the mammary fat pads (MFPs) of BALB/c mice (Figure S3l), and recorded body weight and tumor volume every other day (Figure S3m). Mice were euthanized, and tumors were excised on Day 26 after implantation (Figure S3n). Immunohistochemical staining of serial sections from 4T1‐BALB/c mice demonstrated that BHLHE40 co‐expressed with HIF‐1α (Figure 4k). We also performed immunofluorescence staining in primary human TNBC samples and found that BHLHE40 co‐expressed with BNIP3 (Figure 4l; Table S4). In addition, BHLHE40 mRNA levels were significantly correlated with mRNA‐based hypoxia‐signatures from Winter, Sorensen, Elvidge, Buffa, Ragnum, West, Hu, and Seigneuric (Figure 4m; Figure S3o) [26]. Taken together, these data demonstrate that hypoxia induces whole‐cell BHLHE40 expression in vitro and in vivo through a dual mechanism involving ROS and HIFs, which ensures rapid cellular response during the early stage of hypoxia (Figure 4n).

### 2.5 BHLHE40 Negatively Regulates Intracellular ROS Levels Through Increasing Antioxidant Capacity

To investigate the biological consequence of hypoxia‐induced whole‐cell BHLHE40 expression, we performed RNA‐seq analysis with scrambled control or BHLHE40 knockdown subclones of MDA‐MB‐231 cells exposed to 20% or 1% of O_2_ for 24 h (Figure 5a). Both gene ontology (GO) and Kyoto encyclopedia of genes and genomes (KEGG) enrichment analysis demonstrated the differentially expressed genes (DEGs) between control and BHLHE40 knockdown subclones that were exposed to 1% of O_2_ were enriched in oxidative stress response pathways (Figure S4a,b). Gene set enrichment analysis (GSEA) confirmed impaired antioxidant capacity in BHLHE40‐knockdown cells (Figure S4c). Direct measurement of ROS levels demonstrated that BHLHE40 knockdown increased intracellular ROS levels when cells were exposed to hypoxia for 2 and 24 h (Figure 5b; Figure S5a,b), suggesting a critical role of BHLHE40 in blocking hypoxia‐induced ROS levels.

**FIGURE 5:**
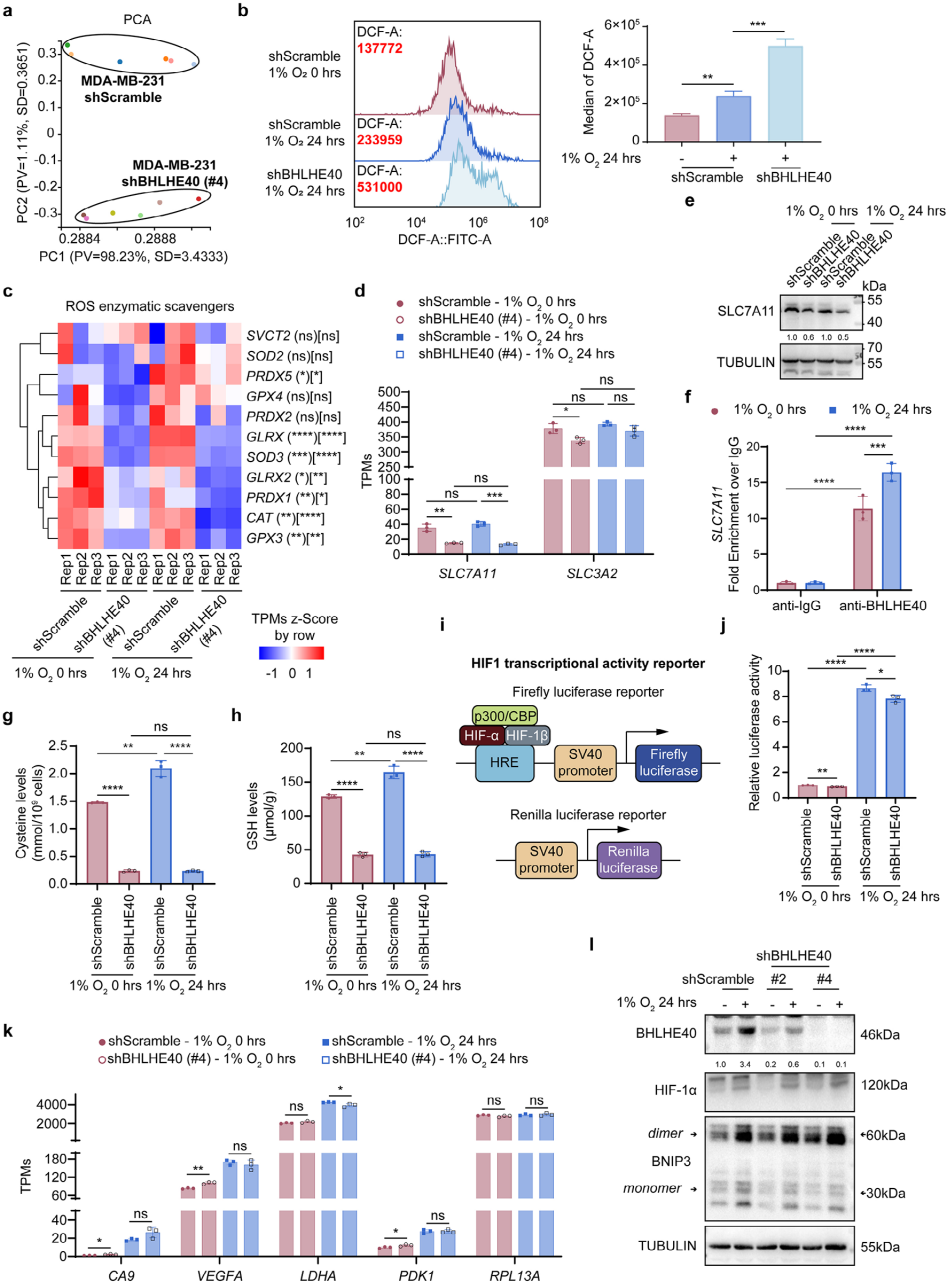
BHLHE40 represses hypoxia‐induced ROS levels through increasing antioxidant capacity without affecting transcriptional activities of HIFs. (a) Principal component analysis (PCA) of gene expression profiles from RNA‐seq of scrambled control and BHLHE40 knockdown subclones of MDA‐MB‐231 cells under 1% of O_2_ for 0 or 24 h. Different color dots, different samples. n = 3. PC, principal component. PV, proportion of variance. SD, standard deviation. (b) Flow cytometry of intracellular ROS levels in scrambled control and BHLHE40 knockdown subclones of MDA‐MB‐231 cells under 1% of O_2_ for 0 or 24 h. DCF, 2',7'‐dichlorofluorescein. A, area. (c) Gene expression heatmap of enzymatic ROS scavengers in scrambled control and BHLHE40 knockdown subclones of MDA‐MB‐231 cells under 1% of O_2_ for 0 or 24 h. Rep, replicate. Parentheses, shBHLHE40 vs. shScramble under 1% of O_2_ for 0 h; brackets, shBHLHE40 vs. shScramble under 1% of O_2_ for 24 h. (d) Expression levels (TPMs) of SLC7A11 and SLC3A2 in scrambled control and BHLHE40 knockdown subclones of MDA‐MB‐231 cells under 1% of O_2_ for 0 or 24 h. (e) Western blot analysis of SLC7A11 proteins in scrambled control and BHLHE40 knockdown subclones of MDA‐MB‐231 cells under 1% of O_2_ for 0 or 24 h. (f) ChIP‐qPCR analysis of BHLHE40 occupancy at the *SLC7A11* promoter in MDA‐MB‐231 cells under 1% of O_2_ for 0 or 24 h. IgG served as a negative control. (g) Cysteine levels in scrambled control and BHLHE40 knockdown subclones of MDA‐MB‐231 cells under 1% of O_2_ for 0 or 24 h. Cysteine levels were normalized to total cell numbers. (h) Reduced glutathione (GSH) levels in scrambled control and BHLHE40 knockdown subclones of MDA‐MB‐231 cells under 1% of O_2_ for 0 or 24 h. GSH levels were normalized to total protein amount. (i) Schematic diagram of HIF transcriptional activity reporter based on Firefly and Renilla luciferase. (j) Dual‐luciferase reporter assay in scrambled control and BHLHE40 knockdown subclones of MDA‐MB‐231 cells transfected with HIF transcriptional activity reporter under 1% of O_2_ for 0 or 24 h. (k) Expression levels (TPMs) of classical HIF‐targeted genes and RPL13A (negative control) in scrambled control and BHLHE40 knockdown subclones of MDA‐MB‐231 cells under 1% of O_2_ for 0 or 24 h. (l) Western blot analysis of BHLHE40, HIF‐1α and BNIP3 proteins in scrambled control and BHLHE40 knockdown subclones of MDA‐MB‐231 cells under 1% of O_2_ for 0 or 24 h. hrs, hours. *p* values were determined by using two‐tailed unpaired Student's t‐tests (c) and one‐way ANOVA (b, d, f, g, h, j, k; n = 3, mean ± SD). ^*^, *p* <0.05; ^**^, *p* <0.01; ^***^, *p* <0.001; ^****^, *p* <0.0001; ns, not significant.

Mechanistically, RNA‐seq analysis demonstrated that BHLHE40 knockdown decreased the expression of several enzymatic ROS scavengers, including SOD3 (superoxide dismutase), PRDX1/5 (peroxidase), GPX3 (glutathione peroxidase), GLRX/GLRX2 (glutaredoxin), and CAT (catalase) (Figure 5c; Figure S5c). Notably, BHLHE40 knockdown markedly decreased the expression of SLC7A11, the catalytic subunit of the cystine transporter, at both mRNA (Figure 5d), total protein (Figure 5e), and plasma membrane (Figure S5d,e) levels. To test whether this reflects direct transcriptional regulation, we performed ChIP‐qPCR under hypoxic conditions in MDA‐MB‐231 cells. We observed significant enrichment of BHLHE40 at the *SLC7A11* promoter relative to IgG control (Figure 5f), indicating direct promoter occupancy. BHLHE40 was also enriched at the *GLRX* promoter (Figure S5f), supporting broad transcriptional activation of antioxidant genes. We also found that BHLHE40 knockdown significantly decreased levels of cysteine (Figure 5g) and reduced glutathione (Figure 5h), the major intracellular antioxidant.

We also investigated whether BHLHE40 regulates cellular redox homeostasis by affecting transcriptional activities of HIFs, and found that BHLHE40 knockdown failed to affect hypoxia‐induced transcriptional activity of HIFs (Figure 5i,j), hypoxia‐induced HIF‐target gene expression (Figure 5k), or hypoxia‐induced HIF‐1α and BNIP3 protein levels (Figure 5l). BHLHE40 knockdown did not lead to significant oxidative damage either, as measured by production of 4‐HNE (Figure S5g). Taken together, these data demonstrate that hypoxia‐induced BHLHE40 negatively regulates intracellular ROS levels by enhancing cellular antioxidant capacity.

### 2.6 BHLHE40 Promotes Hypoxia‐Induced Radioresistance in TNBC

Intratumoral hypoxia is a major reason for resistance to radiotherapy, which kills cancer cells by generating large amounts of ROS [2, 16]. Given the ability of BHLHE40 in inhibiting ROS in response to hypoxia, we investigated the role of BHLHE40 in the regulation of hypoxia‐induced radioresistance in TNBC. Gene expression profiles of radiation sensitivity signatures (RSS) reported by Speers et al. in the TNBC patients from the TCGA_BRCA cohort showed that compared with hypoxia^low^ patients, hypoxia^high^ patients had higher expression of 23 genes positively correlated with radioresistance [40], and lower expression of 28 genes negatively correlated with radioresistance (Figure 6a), confirming that hypoxia mediates radioresistance in TNBC patients. We treated scrambled control or BHLHE40 knockdown subclones of MDA‐MB‐231 (Figure S1a,b) and MDA‐MB‐468 (Figure S6a,b) cells with irradiation at the dose that produced significant killing effect (2 Gy for MDA‐MB‐231 and 4 Gy for MDA‐MB‐468) (Figure S6c,d), and found that BHLHE40 knockdown sensitized hypoxia‐mediated radioresistance, as measured by CCK‐8 and clonogenic assays (Figure 6b,c; Figure S6e). BHLHE40 knockdown also reversed hypoxia‐mediated resistance to irradiation‐induced apoptosis in MDA‐MB‐231 and MDA‐MB‐468 cells (Figure 6d,e). In radioresistant MDA‐MB‐231 and MDA‐MB‐468 cells we generated by continuous irradiation, BHLHE40 protein levels were much higher compared with their parental counterparts (Figure 6f). Rescue experiments in BHLHE40‐knockdown cells showed that wild‐type BHLHE40 restored radioresistance under hypoxia, whereas the C143A and C342A mutants, which fail to undergo the hypoxia‐induced disulfide‐linked dimerization/stabilization, did not rescue the phenotype (Figure S6f).

**FIGURE 6:**
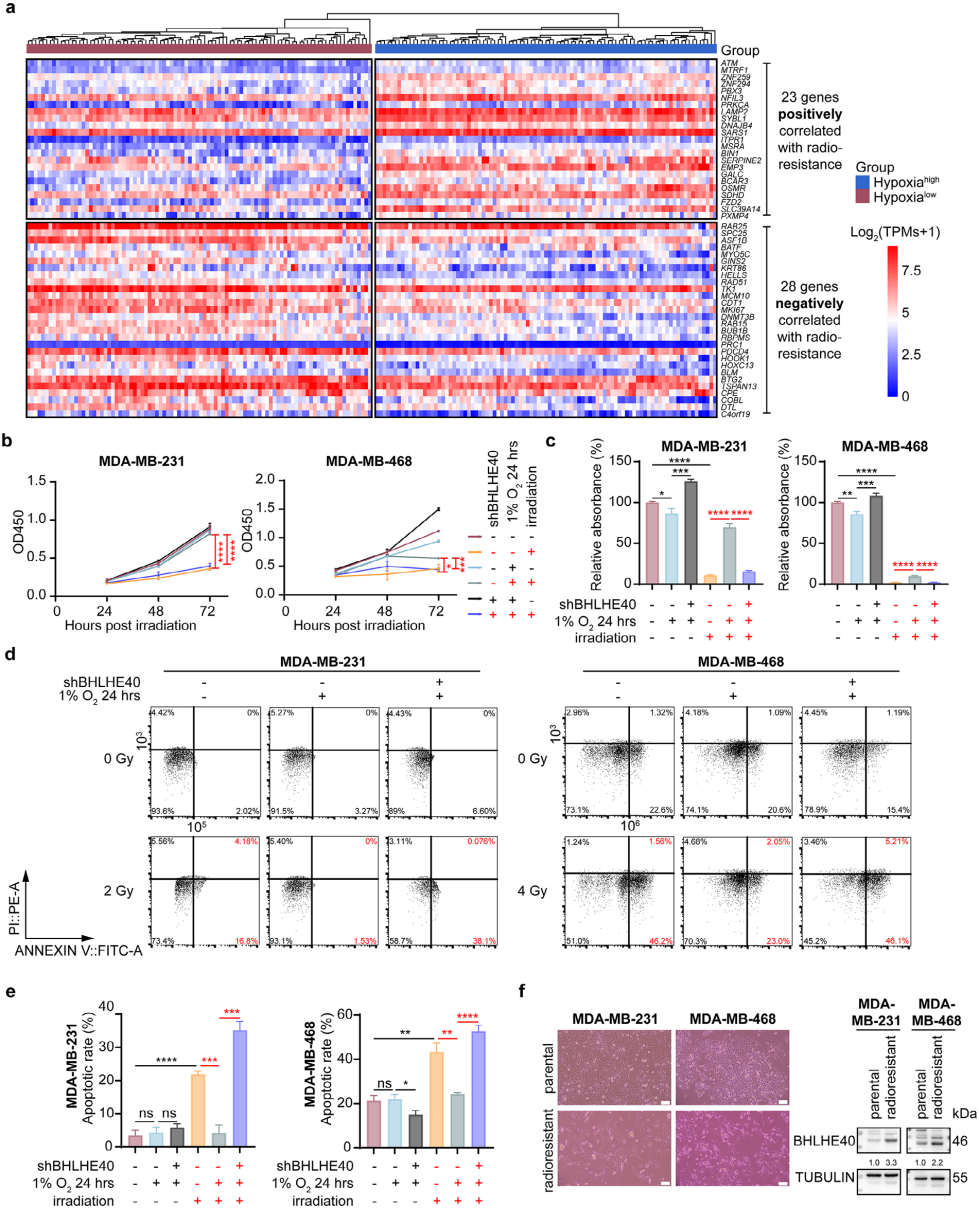
BHLHE40 promotes hypoxia‐induced radioresistance in TNBC in vitro. (a) Gene expression heatmap of radiation sensitivity signatures (RSS) of TCGA_TNBC patients stratified according to our hypoxia‐prognosis model. Row, RSS genes reported by Speers et al. Column, TCGA_TNBC patients. (b) CCK‐8 assay of scrambled control and BHLHE40 knockdown subclones of MDA‐MB‐231 and MDA‐MB‐468 cells under 1% of O_2_ for 0 or 24 h, then 24, 48, or 72 h after exposure to different doses of irradiation. (c) Clonogenic assay of scrambled control and BHLHE40 knockdown subclones of MDA‐MB‐231 and MDA‐MB‐468 cells under 1% of O_2_ for 0 or 24 h, then 14 days after exposure to different doses of irradiation. (d) Flow cytometry of apoptotic rate in scrambled control and BHLHE40 knockdown subclones of MDA‐MB‐231 and MDA‐MB‐468 cells under 1% of O_2_ for 0 or 24 h, then 72 h after exposure to different doses of irradiation. PI, Propidium Iodide. ANNEXIN V, Annexin V‐Phosphatidylserine. (e) Statistical analysis of apoptotic rate including early apoptosis and late apoptosis. (f) Microscopy images of radioresistant and parental counterparts of MDA‐MB‐231 and MDA‐MB‐468 cells (left) and western blot analysis of BHLHE40 proteins (right). Scale bars, 100 µm. hrs, hours. *p* values were determined by using two‐way ANOVA (b; n = 3, mean ± SD), one‐way ANOVA (c, e; n = 3, mean ± SD). ^*^, *p* <0.05; ^**^, *p* <0.01; ^***^, *p* <0.001; ^****^, *p* <0.0001; ns, not significant.

To investigate the role of BHLHE40 in the regulation of hypoxia‐induced radioresistance in TNBC in vivo, we injected scrambled control or BHLHE40 knockdown subclones of MDA‐MB‐231 cells into the MFPs of NOD/SCID mice, and locally irradiated the mice with X‐rays at a single dose of 20 Gy when the tumor volume reached 100 mm^3^. Mice were euthanized, and tumors were excised 21 days after irradiation (Figure 7a,b; Figure S7a). BHLHE40 knockdown significantly sensitized tumor response to radiotherapy, with the tumor growth delay (TGD) prolonged from 4.654 days to 12.181 days (Figure 7c,d).

**FIGURE 7:**
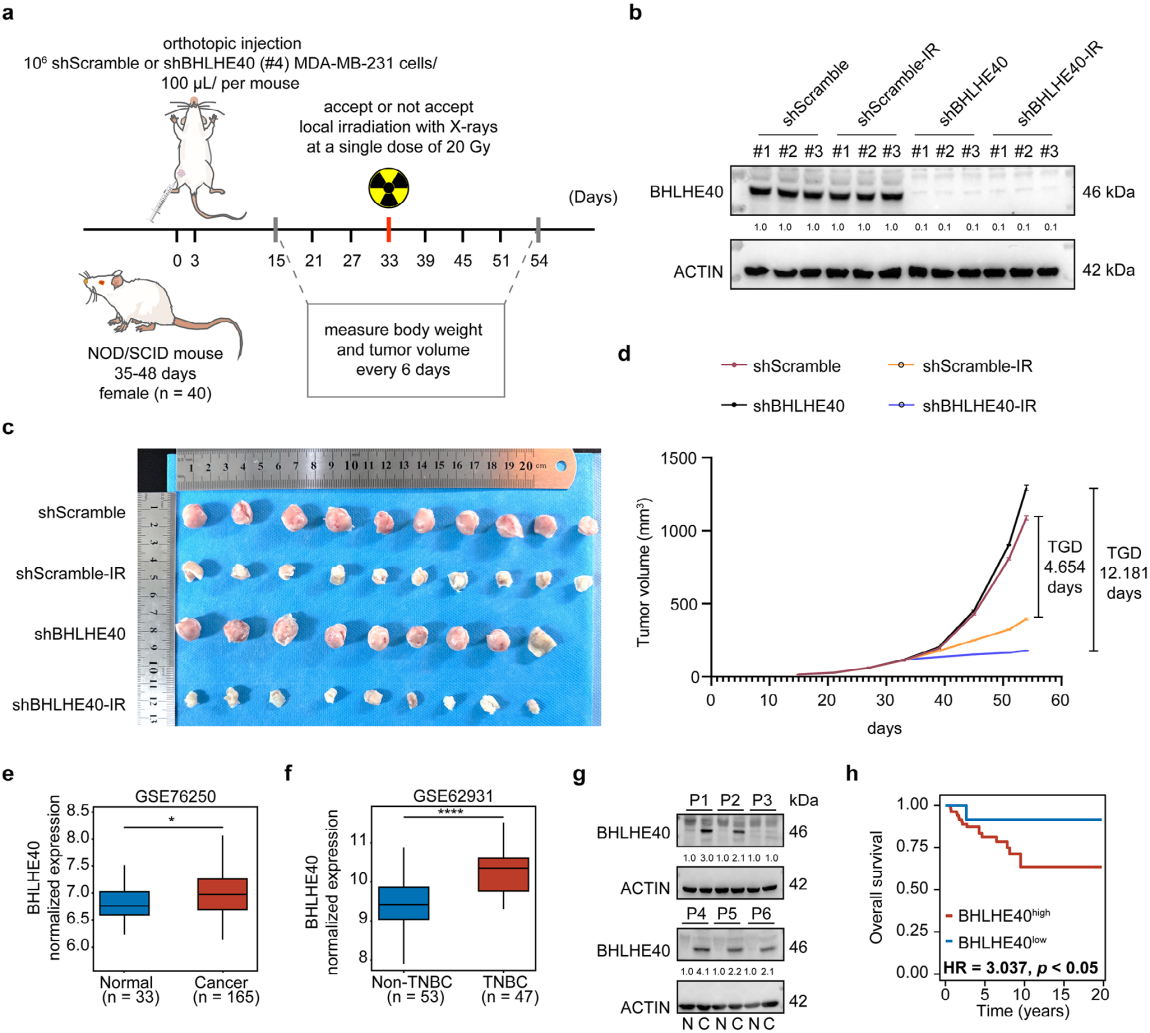
BHLHE40 promotes hypoxia‐induced radioresistance in vivo and is associated with poor prognosis in TNBC. (a) Graphic illustration of experiment plan in the xenograft mouse model. (b) Western blot analysis of BHLHE40 proteins in tumor tissues from mice in different groups. IR, irradiation. (c) Raw image of MDA‐MB‐231‐NOD/SCID mice tumor samples 21 days after irradiation. (d) Tumor volumes of MDA‐MB‐231‐NOD/SCID mice. Data are presented as the mean ± SD (n = 10 in shScramble/n = 9 in shBHLHE40). TGD, tumor growth delay. (e) Expression analysis of BHLHE40 in normal breast epithelium and breast cancer tumor tissue samples in the GSE76250 cohort. n = 33/165, number of samples. (f) Expression analysis of BHLHE40 in non‐TNBC and TNBC tumor tissue samples in the GSE62931 cohort. n = 53/47, number of samples. (g) Western blot analysis of BHLHE40 proteins in primary human TNBC tumor tissue and patient‐matched normal breast epithelium. P, patient. N, normal breast epithelium tissue. C, TNBC tumor tissue. (h) Kaplan–Meier analysis of the overall survival of TCGA_TNBC patients stratified as BHLHE40^high^ and BHLHE40^low^. HR: Hazard Ratio. *p* values were determined by using Wilcoxon tests (e, f) and log‐rank tests (h). ^*^, *p* <0.05; ^****^, *p* <0.0001.

Finally, to explore the clinical implications of our findings, we analyzed BHLHE40 expression from GSE76250 and GSE62931 datasets, and found that BHLHE40 expression was higher in TNBC tumor tissue compared with normal breast epithelium (Figure 7e) [41, 42, 43, 44], and was higher in TNBC compared with non‐TNBC (Figure 7f). Immunoblot analysis demonstrated higher BHLHE40 protein levels in TNBC tissues compared to the patient‐matched normal breast epithelium (Figure 7g; Table S4). Analysis of survival data in TNBC patients from the TCGA_BRCA dataset demonstrated that BHLHE40 expression above the median was associated with decreased overall survival (Figure 7h). Taken together, these data demonstrate that BHLHE40 promotes hypoxia‐induced radioresistance and is associated with poor prognosis in TNBC.

## 3 Discussion

Hypoxia‐induced ROS play a critical role in shaping the intracellular redox homeostasis and the microenvironment of cancer cells [2, 9]. ROS can modulate the function of redox‐sensitive proteins, including some transcription factors, through direct oxidation of cysteine thiol groups and the formation of disulfide bonds [6, 7]. The reaction between ROS and redox‐sensitive transcription factors underscores the significance of ROS in the regulation of gene expression in response to hypoxia‐induced oxidative stress. However, a fundamental paradox is that the compartmentalization of hypoxia‐induced ROS primarily within mitochondria limits their ability to directly interact with nuclear transcription factors [2, 45, 46]. In the present study, we provide a potential solution to this paradox by identifying BHLHE40, a redox‐sensitive transcription factor, as a mitochondrial sensor of hypoxia‐induced ROS. Under hypoxic conditions, mitochondrial BHLHE40 undergoes ROS‐induced oxidation on the thiol groups of cysteine residues, leading to the formation of a disulfide‐linked homodimer. Hypoxia also induces whole‐cell BHLHE40 expression through HIFs‐dependent transcriptional activation. This dual mechanism for modulating BHLHE40 expression ensures rapid elevation of BHLHE40 during the early stage of hypoxia. Functionally, BHLHE40 negatively regulates ROS levels through transcriptional activation of several enzymatic ROS scavengers and SLC7A11. By sensing and inhibiting ROS, BHLHE40 plays a dual and important role to promote hypoxia‐induced radioresistance in TNBC.

Intracellular hypoxia induces complex biological responses, including treatment resistance and mitochondrial production of ROS [2, 3, 4, 6, 7]. It is well‐documented that ROS eradication diminishes the effectiveness of therapies that rely on ROS‐induced cell death, such as radiotherapy, yet the precise regulation remains largely elusive [16]. Therefore, understanding how cancer cells directly sense and regulate ROS under hypoxia is critical for developing strategies to overcome therapeutic resistance. Discoveries of the direct oxidation of cysteine thiol groups in redox‐sensitive transcription factors provide a new perspective on ROS function as a signaling molecule [6, 7, 8], which affects gene expression program and cellular behavior under hypoxic stress [9]. However, a key challenge is the spatial compartmentalization of ROS, which are primarily generated within mitochondria, while transcription factors reside in the nucleus. This challenge is further exacerbated from a biochemical perspective: the highly efficient enzymatic ROS‐scavenging systems are several orders of magnitude faster than the cysteine thiol groups in reaction with ROS [45, 46]. The ROS compartmentalization and low chemical reaction rate with thiol groups lead to the paradox of how ROS can diffuse across the membranes of mitochondria and the nucleus, without being eradicated by the highly abundant and efficient enzymatic ROS scavengers located in the cytoplasm. Our findings address this challenge by demonstrating that the redox‐sensitive transcription factor BHLHE40 is present in the mitochondria, providing a spatial advantage to directly sense and interact with ROS (Figure 2a–d, Figure 3a,b,f, Figure 4b; Figure S2f). Therefore, BHLHE40 proteins can be oxidized by high local concentrations of ROS and bypass the biochemical limitations imposed by enzymatic ROS‐scavenging systems. It is noteworthy that the predominant nuclear BHLHE40 staining observed in human TNBC tissue and the mitochondrial localization detected by immunoelectron microscopy and subcellular fractionation are complementary rather than contradictory. As a transcription factor, BHLHE40 is expected to be most abundant in the nucleus, particularly in hypoxic tumor regions where it participates in gene regulation. By contrast, the mitochondria‐localized BHLHE40 represents a smaller but functionally critical pool where ROS sensing occurs.

One caveat of our study is that the contribution of mitochondrial BHLHE40 in mediating radioresistance is not fully elucidated. Although we have demonstrated that wild‐type, but not C143A/C342A mutant, BHLHE40 restored radioresistance in BHLHE40‐knockdown cells, we did not generate a mitochondria‐targeted or nucleus‐restricted BHLHE40 mutant to conclude that mitochondrial localization is the exclusive spatial determinant of all downstream phenotypes. In addition, the mechanistic relationship between hypoxia‐induced mitochondrial BHLHE40 stabilization and whole‐cell upregulation remains unclear. The mitochondrial BHLHE40 may serve as a source for the nuclear pool of BHLHE40, but we have not directly visualized the trafficking of oxidized BHLHE40 to the nucleus nor tested how its redox state modulates nuclear transcriptional activity. Future work will elucidate the contribution of mitochondrial BHLHE40 in mediating radioresistance in vitro and in vivo, and examine potential hypoxia‐dependent shuttling between mitochondria and nucleus, thereby establishing how its ROS‐sensing function relates to nuclear activity.

Our results demonstrate that BHLHE40 mediates hypoxia‐induced radioresistance in TNBC, and the implications of these findings extend beyond radiotherapy resistance. ROS play a pleiotropic role in cancer, acting as both signaling molecules and cytotoxic agents [6, 7, 9]. Several therapeutic strategies are designed to exploit the vulnerability of cancer cells to oxidative stress [9, 16, 47]. However, the regulatory role of BHLHE40 in upregulating enzymatic ROS‐scavenging systems presents a challenge to the efficacy of therapeutic strategies that enhance cytotoxic ROS. By maintaining ROS levels below cellular tolerance, BHLHE40 mitigates the cytotoxic effects of therapies that rely on ROS‐induced cell death, such as radiotherapy. Therefore, targeting BHLHE40 may enhance the effectiveness of ROS‐dependent therapies by disrupting the protective mechanisms that shield TNBC cells from oxidative stress, especially under tumor hypoxia.

In summary, our study identifies BHLHE40 as a key regulator of hypoxia‐induced ROS and radioresistance in TNBC. BHLHE40 is localized in the mitochondrial matrix and functions as a sensor of hypoxia‐induced ROS via the formation of disulfide bonds between cysteine residues. Our study provides compelling evidence that targeting BHLHE40 represents a promising strategy to overcome hypoxia‐mediated radioresistance. Future studies should explore the clinical relevance of these findings and investigate strategies to target BHLHE40 in combination with existing ROS‐dependent therapeutic approaches to enhance patient outcomes in TNBC.

## 4 Conclusion

Hypoxia‐induced BHLHE40 plays a dual role in promoting radioresistance in TNBC. It serves not only as a sensor but also as a negative regulator of ROS. Our study demonstrates that the early stage of hypoxia triggers a dual mechanism for rapid BHLHE40 upregulation: ROS‐dependent post‐translational modifications and HIF‐dependent transcriptional activation. BHLHE40 experiences ROS‐induced oxidation of cysteine thiol groups and forms a disulfide‐linked homodimer in mitochondria. BHLHE40 homodimer induces expression of several enzymatic ROS scavengers and cystine transporter SLC7A11 through transcriptional activation, which reduces ROS levels and promotes radioresistance in TNBC.

## 5 Experimental Section

### 5.1 Bioinformatics Analysis

The TCGA_BRCA dataset that comprised 187 TNBC patients with RNA‐seq data and clinical information was accessed from The Cancer Genome Atlas (TCGA) (https://tcga‐data.nci.nih.gov/tcga/). The HALLMARK_HYPOXIA gene set that consisted 200 hypoxia‐upregulated genes was accessed from the MSigDB (https://www.gsea‐msigdb.org/gsea/msigdb) [25]. The univariate Cox regression analysis was performed by using *R. survival* package. The LASSO regression algorithm was applied by using *R. glmnet* package. The hypoxia scores were calculated based on the 7 hypoxia‐prognosis‐related genes, weighted using the LASSO coefficients as follows: hypoxia score = Σ β_n_ of gene_n_^*^ expression of gene_n_, where β is the LASSO coefficient (Table S1). Eight independent hypoxia signatures were accessed from a previous publication [26]. The Pearson correlation analysis of hypoxia scores was performed by using *R. cor. test* and *R. circlize* packages. GSE103091 that comprised 107 TNBC patients with RNA‐seq data and clinical information [27, 28], GSE111653 that contains RNA‐seq data of 32 breast cancer cells or normal human mammary epithelial cells exposed to 20% or 1% of O_2_ for 24 h [29, 30], GSE76250 that comprised RNA‐seq data of 33 normal breast epithelium and 165 breast cancer tumor tissue [41, 42, 43], and GSE62931 that comprised RNA‐seq data of 53 non‐TNBC tumor tissue and 47 TNBC tumor tissue [44], were accessed from the Gene Expression Omnibus (GEO) (https://www.ncbi.nlm.nih.gov/geo/). The receiver operating characteristic (ROC) analysis was performed by using *R. pROC* package. The Kaplan‐Meier analysis was performed by using *R. median, R. survival and R. survminer* packages. The principal component analysis, GO and KEGG enrichment analysis, and GSEA were performed in the Dr. Tom Data Visualisation Solution (BGI Genomics Co., Ltd). The RSS gene set that consisted 23 genes previously reported to be positively correlated with radioresistance and 28 genes previously reported to be negatively correlated with radioresistance was accessed from a previous publication [40].

### 5.2 Cell Culture and Reagents

MDA‐MB‐468 (RRID: CVCL_0419), MDA‐MB‐231 (RRID: CVCL_0062), HS578T (RRID: CVCL_0332), MCF7 (RRID: CVCL_0031) and 293T (RRID: CVCL_0063) cells were cultured in high glucose DMEM (VivaCell‐C3113, Shanghai, China); SUM159 (RRID: CVCL_5423) cells were cultured in DMEM/F12 (VivaCell‐C3130); 4T1 (RRID: CVCL_0125) cells were cultured in RPMI1640 (VivaCell‐C3010), all of which were supplemented with 10% fetal bovine serum (VivaCell‐C04001) and 1% penicillin‐streptomycin (Solarbio‐P1400, Beijing, China). Cells were maintained at 37°C in a 5% CO_2_, 95% air incubator. All cells were obtained from the Shanghai Zhong Qiao Xin Zhou Biotechnology Co., Ltd (China) on August 2021. All cells were tested negative for mycoplasma contamination, and all human cells were authenticated using short tandem repeat (STR) profiling.

For hypoxia treatment, cells were maintained at 37°C in a modular incubator chamber (STEMCELL‐27310, Vancouver, CA) flushed with a gas mixture containing 5% CO_2_, 1% O_2,_ and 94% N_2_ for the indicated time.

NAC (MCE‐HY‐B0215, New Jersey, USA) was dissolved in water and used at the final concentration of 0.5 mM. H_2_O_2_ (Sigma‐H1009, Michigan, USA) was used at the final concentration of 0.2 mM. VH298 (MCE‐HY‐100947) was dissolved in DMSO and used at the final concentration of 0.1 mM. MG‐132 (MCE‐HY‐13259) was dissolved in DMSO and used at the final concentration of 0.1 mM. DTT (MCE‐HY‐15917) was dissolved in water and used at the final concentration of 2 mM.

### 5.3 Immunoelectron Microscopy

MDA‐MB‐468 cells and scramble or BHLHE40 knockdown subclone of MDA‐MB‐231 cells were fixed with a mixture of 4% paraformaldehyde and 0.05% glutaraldehyde in 0.15 M HEPES buffer for 5 min at room temperature (RT) and then replaced with 4% paraformaldehyde in 0.15 m HEPES buffer for 30 min at RT, followed by washing with PBS for six times at RT and incubation with blocking solution for 30 min at RT. Cells were incubated with primary antibody (Table S5) diluted in blocking solution overnight at 4°C. On the following day, cells were washed with PBS for six times at RT and incubated with goat anti‐rabbit Fab’ fragments coupled to gold particles (diluted in blocking solution 1: 100) for 2 h and washed with PBS for six times at RT. Meanwhile, the activated GoldEnhance EM (Nanoprobes‐2113, New York, USA) was prepared according to the manufacturer's instructions, and 100 µL of the activated GoldEnhance EM were added into each sample. The reaction was monitored under a conventional light microscope and was stopped after 5–10 min when the cells had turned “dark enough” by washing several times with PBS. Cells were incubated with a 1: 1 mixture of 2% osmium tetraoxide in distilled water and 3% potassium ferrocyanide in 0.2 m sodium cacodylate (pH 7.4) for 1 h at RT and then rinsed with PBS for six times and then with distilled water. The samples were dehydrated in graded ethanol (50%, 70%, 90%, 100%, 3 × 10 min each) and were subsequently incubated in 1: 1 mixture of 100% ethanol and Epoxy resin for 2 h at RT. The mixture was then removed with a pipette, and finally the samples were embedded in Epoxy resin for 2 h at RT. The resin was polymerized in an oven for at least 10 h at 60°C [48, 49]. Sections were analyzed with a Tecnai 20 High Voltage EM operating at 200 kV (FEI, Oregon, USA).

### 5.4 Immunofluorescence (IF)

For cell IF assay, cells were grown on glass coverslips placed inside 24‐well plates. After the indicated treatment, cells were fixed with 4% paraformaldehyde for 1 h at RT and then permeabilized with 0.4% Triton X‐100 (Solarbio‐T8200) for 15 min at RT, and blocked with 10% BSA‐V (Solarbio‐A8020) for 2 h at RT. Then, cells were incubated with primary antibody (Table S5) diluted in a 1: 4 mixture of 10% BSA‐V and 0.4% Triton X‐100 overnight at 4°C. On the following day, the cells were washed with PBS for three times at RT and incubated with secondary antibodies (Alexa Fluor Plus 488, Invitrogen‐A32731; Alexa Fluor Plus 568, Invitrogen‐A10037) (California, USA) (diluted in primary antibody dilution 1: 200) for 1 h and washed with PBS for three times at RT. Finally, the samples were covered in antifade mounting medium with DAPI (Beyotime‐P0131, Shanghai, China). The IF staining images were taken on LSM980 (Zeiss, Oberkochen, GER) and analyzed with ImageJ software.

For tissue IF assay, fresh tissues were fixed with 4% paraformaldehyde for overnight at 4°C and then dehydrated by graded sucrose (15% sucrose for overnight; 30% sucrose for overnight; 30% sucrose for overnight) at 4°C. Tissues were embedded in O.C.T. compound (Sakura Tissue‐Tek‐4583, California, USA) at −20°C and cut into frozen sections. The subsequent steps were the same as those in the cell IF assay.

### 5.5 Mitochondrial Protease Protection Assay

Mitochondria were isolated using the Mitochondria Isolation Kit (Proteintech‐PK10016, Illinois, USA) and resuspended in either mitochondrial isolation or swelling buffer, containing 10 mM HEPES‐KOH (pH7.4) and 1 mM EDTA. For proteinase K treatment, a final concentration of 20 µg mL^−1^ proteinase K (Sigma–Aldrich‐3115828001, Missouri, USA) was applied, with or without 1% Triton X‐100. This mixture was then subjected to incubation on ice for 15 min, after which 4 mM PMSF was introduced to halt proteinase K activity. To achieve complete protein denaturation, all samples were exposed to a 100 °C metal bath for 10 min before undergoing SDS−PAGE analysis.

### 5.6 CCK‐8 Assay

Cells were grown in 96‐well plates. Following the indicated treatment, each well was replaced with fresh medium containing 10% CCK‐8 reagent (MCE‐HY‐K0301). After incubation for 1 h at 37°C, the absorbance of each well was quantified at the wavelength of 450 nm using INFINITE 200 PRO (TECAN, California, USA).

### 5.7 Flow Cytometry

Following the indicated treatment, cells were dissociated from the plates with trypsin for 3 min at 37°C and then passed through a 40 µm filter to generate single‐cell suspensions. For intracellular ROS levels, single‐cells were stained using DCFH‐DA (Sigma‐D6883) for 30 min at 37°C and detected via FITC channel. For apoptotic rate, single‐cell was stained using FITC Annexin V Apoptosis Detection Kit I (BD‐556547, New Jersey, USA) according to the manufacturer's instructions and detected via FITC and PE dual channels. For cell‐surface xCT/SLC7A11 analysis, non‐permeabilized single cells were incubated with anti‐SLC7A11 antibody (Table S6) followed by fluorescent secondary antibody and analyzed by flow cytometry. Flow cytometry was performed using CytoFLEX (Beckman Coulter, California, USA), and data analysis were performed with FlowJo software.

### 5.8 Non‐Reducing and Reducing Western Blot Analysis and Immunoprecipitation (IP)

For western blot analysis, whole‐cell proteins and mitochondrial proteins were extracted with RIPA lysis buffer (50 mM Tris, pH 7.4, 150 mM NaCl, 1% Triton X‐100, 1% sodium deoxycholate, 1% SDS, et al., supplemented with protease inhibitor cocktail). Mitochondria were isolated using Mitochondria Isolation Kit (Proteintech‐PK10016), nuclear and cytoplasmic proteins were isolated using Nuclear and Cytoplasmic Protein Extraction Kit (Beyotime‐P0027), according to the manufacturer's instructions.

For non‐reducing western blot analysis, the proteins were split into two aliquots per sample. An appropriate amount of Non‐reducing SDS‐PAGE Sample Loading Buffer without DTT and β‐mercaptoethanol (β‐ME) (Beyotime‐P0016P) was added to one aliquot for non‐reducing western blot analysis, and an appropriate amount of SDS‐PAGE Sample Loading Buffer with DTT (Beyotime‐P0015) was added to the other aliquot for reducing western blot analysis. All samples were boiled for 10 min at 100°C before western blot analysis except for the samples for detecting cell membrane protein SLC7A11, which were incubated for 30 min at 37°C.

The proteins were separated by SDS‐PAGE, blotted onto nitrocellulose membranes, and incubated with primary antibodies (Table S6) for overnight at 4°C. On the following day, the membranes were incubated with secondary antibodies conjugated with HRP (goat anti‐rabbit IgG, ZSGB‐BIO‐ZB2301; goat anti‐mouse IgG, ZSGB‐BIO‐ZB2305) (Beijing, China) (diluted in PBS‐T 1: 2000) for 1 h at RT, and chemiluminescent signal was detected using Immobilon Western Chemiluminescent HRP Substrate (Merck Millipore‐WBKLS0100, Massachusetts, USA).

For IP assay, mitochondrial proteins were extracted with western and IP lysis buffer (20 mM Tris, pH 7.5, 150 mM NaCl, 1% Triton X‐100, et al., supplemented with protease inhibitor cocktail). The proteins were incubated with the indicated antibodies (Table S6) for overnight at 4°C. The protein‐antibody complexes were subjected to precipitation with Protein A/G magnetic beads (Selleck‐B23202, Texas, USA) for 2 h at RT, washed with the above‐mentioned western and IP lysis buffer for five times, eluted with an appropriate amount of Non‐reducing SDS‐PAGE Sample Loading Buffer without DTT and β‐ME (Beyotime‐P0016P), and boiled for 10 min at 100°C for non‐reducing western blot analysis.

### 5.9 Ellman's Assay

For abovementioned isolated mitochondrial proteins, after immunoprecipitating and washing, the protein‐antibody complexes were eluted with an appropriate amount of elution buffer (0.1 m glycine, 0.02 M hydrochloric acid, pH 3.0). For total cysteine thiols analysis, the samples were denatured and reduced using Protein Cysteine Detection Kit (Beyotime‐S0145) according to the manufacturer's instructions; for total free thiols analysis, the samples were denatured using Total Thiol Detection Kit (Beyotime‐S0141) according to the manufacturer's instructions. The contents of disulfide bonds were calculated as the contents of total cysteine thiols minus the contents of total free thiols. The absorbance of each sample was quantified at the wavelength of 412 nm using INFINITE 200 PRO (TECAN). Levels of total cysteine thiols and total free thiols, and contents of disulfide bonds were normalized to protein concentration after immunoprecipitation.

### 5.10 Lentivirus Transduction

pLKO.1 puro lentiviral backbones for cloning and expression of shRNA sequences were purchased from addgene (8453) (Massachusetts, USA), and all shRNA sequences are shown in Table S8. Lentiviruses were packaged in 293T cells, and viral supernatant was collected 48 h after transfection. TNBC cells were transduced with viral supernatant supplemented with 10 µg mL^−1^ polybrene (Solarbio‐H8761). After 24 h, cells were maintained with fresh medium containing 0.5 µg mL^−1^ puromycin (Beyotime‐ST551) for selection of stably transfected clones.

### 5.11 Transfection

BHLHE40 overexpressing plasmid with site‐specific mutations was purchased from Jinan Boshang Biotechnology Co., Ltd (CN). DNA sequencing was performed to verify the mutated gene sequences. For transfection, plasmids were transfected into cells with LipofectamineTM 3000 Transfection Reagent (ThermoFisher‐L3000015) according to the manufacturer's instructions.

### 5.12 Reverse Transcription and qPCR

Total RNA was extracted with TRIzol (ThermoFisher‐15596026CN, Massachusetts, USA), precipitated with isopropanol, and treated with DNase I, RNase‐free (ThermoFisher‐EN0521). The concentration of RNA samples was measured with a NanoDrop microvolume spectrophotometer (ThermoFisher), and the integrity of RNA samples was checked by agarose gel electrophoresis. cDNA synthesis was performed using the HiFiScript cDNA Synthesis Kit (CWBIO‐CW2569M, Taizhou, China). qPCR analysis was performed using UltraSYBR Mixture (CWBIO‐CW0957H) and detected using the CFX Connect Real‐Time System (BIO‐RAD, California, USA). The expression of each target mRNA relative to 18S rRNA was calculated based on the cycle threshold (Ct) as the expression = 2^−Δ(ΔCt)^, in which ΔCt = Ct (target gene)—Ct (18S rRNA), and Δ(ΔCt) = ΔCt (test sample)—ΔCt (control sample). All primer sequences (5’ to 3’) for RT‐qPCR are shown in Table S7.

### 5.13 Chromatin Immunoprecipitation (ChIP) Assay

ChIP assay was conducted using a SimpleChIP Plus Sonication Chromatin IP Kit (CST‐56383, Massachusetts, USA) following the manufacturer's protocols. Specifically, cells were fixed with 1% formaldehyde and subsequently quenched with 0.125 M glycine. The cells were sonicated using a Bioruptor Pico sonication device. DNA was then immunoprecipitated using either control IgG or anti‐BHLHE40 antibody, followed by qPCR analysis. All primer sequences (5’ to 3’) for ChIP‐qPCR are shown in Table S7.

### 5.14 Animal Experiments

All animal experiments were performed according to the protocols approved by the Institutional Animal Care and Use Committee of Cheeloo College of Medicine, Shandong University (ECSBMSSDU2021‐2‐116). 35‐ to 48‐day‐old female BALB/c mice and NOD/SCID mice were purchased from Beijing Vital River Laboratory Animal Technology Co., Ltd (China). For orthotopic injection of BALB/c mice, 10^6^ 4T1 cells/ 100 µL were injected into the MFPs of the mice. Mouse body weight and tumor size were measured every other day, and mice were euthanized with tumor volume exceeding 1000 mm^3^. For orthotopic injection of NOD/SCID mice, 10^6^ scrambled control or BHLHE40 knockdown subclone of MDA‐MB‐231 cells/ 100 µL were injected into the MFPs of the mice. Mouse body weight and tumor size was measured every 6 days. When the tumor volume reached 100 mm^3^, mice were locally irradiated with X‐rays at a single dose of 20 Gy and were euthanized 21 days after irradiation. Tumor volume was calculated using the formula: tumor volume = 0.5 × L × W^2^, where L is the longest dimension, and W is the perpendicular dimension.

### 5.15 Immunohistochemistry (IHC)

For tissue IHC assay, fresh tissues were fixed with 4% paraformaldehyde for overnight at 4°C and then dehydrated by graded sucrose (15% sucrose for overnight; 30% sucrose for overnight; 30% sucrose for overnight) at 4°C. Tissues were embedded in O.C.T. compound (Sakura Tissue‐Tek‐4583) at −20°C and cut into frozen sections. The frozen sections were incubated with 3% H_2_O_2_ (Sigma‐H1009) for 15 min at RT, permeabilized with 0.4% Triton X‐100 (Solarbio‐T8200) for 15 min at RT, and blocked with 10% BSA‐V (Solarbio‐A8020) for 2 h at RT. Then, the frozen sections were incubated with primary antibody (Table S5) diluted in a 1: 4 mixture of 10% BSA‐V and 0.4% Triton X‐100 for overnight at 4°C. On the following day, the frozen sections were washed with PBS for three times at RT and incubated with secondary antibodies (ZSGB‐BIO‐PV‐9000) at RT according to the manufacturer's instructions. DAB staining was performed using DAB Staining Kit (ZSGB‐BIO‐ZLI‐9018) according to the manufacturer's instructions. The IHC staining images were taken on a Panoramic Scanning Microscope (Olympus, Tokyo, Japan) and analyzed with ImageJ software.

### 5.16 Human Samples

Human sample study protocols and sample use were approved by the Medical Ethics Review Committee of Cheeloo College of Medicine, Shandong University (ECSBMSSDU2024‐1‐61). All experiments were carried out in conformity to the principles set out in the WMA Declaration of Helsinki. Informed written consent for possible scientific research was provided by all patients before the surgery operation began. All human sample information is shown in Table S4.

### 5.17 Cysteine and Reduced Glutathione (GSH) Measurement

Following the indicated treatment, intracellular cysteine levels were measured using Cysteine Colorimetric Assay Kit (Elabscience‐E‐BC‐K352‐M, Wuhan, China) according to the manufacturer's instructions and the absorbance of each sample was quantified at the wavelength of 600 nm using INFINITE 200 PRO (TECAN); intracellular GSH levels were measured using Reduced Glutathione Colorimetric Assay Kit (Elabscience‐E‐BC‐K030‐M) according to the manufacturer's instructions and the absorbance of each sample was quantified at the wavelength of 405 nm using INFINITE 200 PRO (TECAN).

### 5.18 Dual Luciferase Reporter Assay

MDA‐MB‐231 cells stably transfected with HIF transcriptional activity reporter plasmid pHRE‐SV‐Firefly and control plasmid pSV‐Renilla were transduced with BHLHE40 shRNA viral supernatant supplemented with 10 µg mL^−1^ polybrene (Solarbio‐H8761). 1 × 10^4^ abovementioned MDA‐MB‐231 cells were seeded in each well of 96‐well plates. Following the indicated treatment, the ratio of Firefly/Renilla luciferase activity was determined by using the Dual‐Luciferase Reporter Assay System (Promega‐E1960, Wisconsin, USA) according to the manufacturer's instructions and detected using an LB960 microplate luminometer (Berthold, Madison, GER).

### 5.19 Clonogenic Assay

Following the indicated treatment, cells were grown in six‐well plates. After culturing for 14 days, cells were fixed with 4% paraformaldehyde for 1 h at 4°C, stained with crystal violet (Beyotime‐C0121), and counted using ImageJ software. Relative absorbance was calculated as the percentage of the control sample.

### 5.20 Development of Radioresistant TNBC Cells

The radioresistant MDA‐MB‐231 and MDA‐MB‐468 derivative cells were developed by continuous irradiation by using a cabinet X‐ray system (Precision X‐ray, Connecticut, USA). Parental MDA‐MB‐231 and MDA‐MB‐468 cells were irradiated at a dose of 2 Gy every other day for 7 weeks, with a total dose of 50 Gy. The surviving clones were expanded and considered as radioresistant cells.

### 5.21 Statistical Analysis

For RNA‐seq data, gene expression levels were quantified and normalized as TPMs. Data are presented as the mean ± SD, with the exact sample size (n) for each statistical analysis indicated in the corresponding figure legends. For two‐group comparisons, two‐tailed unpaired Student's t‐test and Wilcoxon test were applied. For multiple group comparisons, one‐ or two‐way ANOVA with Tukey's post‐hoc test were applied. Survival analysis was performed using univariate Cox regression and the log‐rank test. Correlations were evaluated using Pearson correlation analysis. Statistical significance was set at *p* <0.05. All statistical analyses were performed using *R package* or GraphPad Prism software.

## Author Contributions

J. L. and H. L. designed the research study. J. L., Z. N., X. C., G. J., and Y. Z. performed experiments and acquired data. J. L. and Z. Z. performed bioinformatics analysis. J. L., Z. N., Y. Z., X. D., Z. Z., J. L., Y. Y., F. S. and Z. Y. performed statistical analysis. J. L., Z. N. and H. L. analyzed the data. J. L. and H. L. wrote the manuscript. H. L. supervised the study. All authors reviewed and commented on the manuscript.

## Funding

This work was supported by National Natural Science Foundation of China (Overseas Excellent Young Scientist Fund Program to H.L.; 82473126 to H.L.), China Association for Science and Technology Youth Talent Support Program for Doctoral Students (156‐O‐180‐0000689‐2 to J.L.), Natural Science Foundation of Shandong Province (ZR2025LMB001 to H.L.; ZR2021YQ50 to H.L.; ZR2022QC212 to G.J.), Shandong Postdoctoral Science Foundation (SDZZ‐ZR‐202501286 to J.L.), Cutting Edge Development Fund of Advanced Medical Research Institute at Shandong University (GYY2023QY01 to H.L.), and Young Student Basic Research Project Cultivation Program of Shandong University (SDU‐QM‐Y2024017 to J.L.). Haiquan Lu is a Taishan Scholar Young Talent Professor of Shandong Province and Distinguished Young Professor at Shandong University.

## Ethics Statement

Human sample study protocols and sample use were approved by the Medical Ethics Review Committee of Cheeloo College of Medicine, Shandong University (ECSBMSSDU2024‐1‐61). All experiments were carried out in conformity to the principles set out in the WMA Declaration of Helsinki. Informed written consent for possible scientific research was provided by all patients before the surgery operation begins. All animal experiments were performed according to the protocols approved by the Institutional Animal Care and Use Committee of Cheeloo College of Medicine, Shandong University (ECSBMSSDU2021‐2‐116).

## Conflicts of Interest

The authors declare no conflicts of interest.

## Supporting information




**Supporting File 1**: advs76864‐sup‐0001‐SuppMat.docx.

**Supporting File 2**: advs76864‐sup‐0002‐FigureS1‐S7.zip.

## Data Availability

The data that support the findings of this study are available from the corresponding author upon reasonable request.
