# Supplementary material for: Intratumoral Hypoxia Triggers Mitochondrial BHLHE40 ROS Sensing Pathway to Promote Radioresistance in Triple‐Negative Breast Cancer
Source: Adv Sci (Weinh). 2026 Jul 27;13(59):e76864. doi: 10.1002/advs.76864 (PMC13403735; doi:10.1002/advs.76864)
Supplement: Supplementary file 1 [file ADVS-13-e76864-s002.docx]

Supporting Information

Intratumoral Hypoxia Triggers Mitochondrial BHLHE40 ROS Sensing Pathway to Promote Radioresistance in Triple-Negative Breast Cancer

*Jia Liu, Ziliang Nie, Xi Chen, Guangyu Ji, Yajing Zhang, Zhiqun Zhao, Yuhong Zhang, Xinlong Du, Zhenzhen Zhou, Jiayi Li, Yaozong Yang, Fengqi Sun, Zhibo Yan, and Haiquan Lu**

*J. Liu and Z. Nie contributed equally to this work.*

Supporting Information includes:

Figure S1 to S7

Table S1 to S9

Tumor weight data of Fig. 7c & Fig. S3n

Original Western Blots-1 to -5

Microscope Raw Images-1 to -4

Figure S1 Supplementary figures of Figure 2

(a) RT-qPCR analysis of BHLHE40 mRNA levels in scramble control and BHLHE40 knockdown subclones of MDA-MB-231 cells.
(b) Western blot analysis of BHLHE40 proteins in scramble control and BHLHE40 knockdown subclones of MDA-MB-231 cells.

#2, #4, different BHLHE40 shRNA sequences. *p* values were determined by using one-way ANOVA (a; n = 3, mean ± SD). ***, *p* < 0.001; ****, *p* < 0.0001.

Figure S2 Supplementary figures of Figure 3

(a) Schematic diagram of BHLHE40 homodimer structure. BHLHE40 homodimer has transcriptional activity. N, N terminus. Basic, basic domain that allows DNA binding. Helix-loop-helix (HLH), HLH domain that allows the formation of homodimer or heterodimer. Orange, orange domain that may confer specificity of function to different family members and/or may be involved in dimerization. Pro-rich, proline-rich domain. C, C terminus.
(b) The positions of eight cysteine residues in the BHLHE40 domain structure. C, cysteine. BHLH, basic helix-loop-helix domain. Orange, orange domain. 1 to 412, N terminus to C terminus.
(c) The hypothesis that mitochondrial BHLHE40 functions as a ROS sensor through the formation of disulfide-linked homodimer. Cys, cysteine. -SH, thiol. -S-S-, disulfide bond.
(d) CCK-8 assay of MDA-MB-231 and MDA-MB-468 cells under 1% of O_2_ for 0 or 2 hours with or without 0.5 mM NAC or 0.1 mM VH298 pre-treatment.
(e) Flow cytometry of intracellular ROS levels in MDA-MB-231 and MDA-MB-468 cells under 1% of O_2_ for 0 or 2 hours with or without 0.5 mM NAC pre-treatment. DCF, 2',7'-dichlorofluorescein. A, area.
(f) Non-reducing and reducing western blot analysis of BHLHE40 proteins in BHLHE40 knockdown subclones of MDA-MB-468 cells transfected with WT or mutant BHLHE40 expression vectors, under 1% of O_2_ for 0, 2 or 24 hours with or without 0.5 mM NAC pre-treatment. Red arrows, BHLHE40 homodimers. Black arrows, BHLHE40 monomers. NAC, N-acetyl-cysteine.
(g) Disulfide bond prediction using AlphaFold 3. Red boxes, the potential amino acid residues to form intermolecular disulfide bond. C, cysteine. 3.4, the distance between two cysteine residues.
(h) Flowchart to test the effect of different cysteine mutations of BHLHE40 protein on response to hypoxia and ROS. #2, #4, different BHLHE40 shRNA sequences. WT, wild-type. KD, knockdown. MUT, mutant.
(i) DNA sequences confirming the site-specific mutations of BHLHE40 (C13A, C71A, C143A, C149A, C219A, C260A, C322A and C342A). C, cysteine. A, alanine.

hrs, hours. *p* values were determined by using one-way ANOVA (d, e; n = 3, mean ± SD). *, *p* < 0.05; **, *p* < 0.01; ***, *p* < 0.001; ns, not significant.

Figure S3 Supplementary figures of Figure 4

(a) Western blot analysis of BHLHE40 proteins in the whole cell lysates and cytoplasmic lysates of MDA-MB-468 cells under 1% of O_2_ for 0, 2, or 24 hours. Tubulin, cytoplasmic protein controls. Lamin B1, nuclear protein control. WCL, whole-cell lysates. CYTO, cytoplasmic lysates.
(b) Western blot analysis of BHLHE40 proteins in HS578T, SUM159 and MCF7 cells under 1% of O_2_ for 0, 2, or 24 hours.
(c) Western blot analysis of BHLHE40, HIF-1α and HIF-1β proteins in MDA-MB-468 cells under 1% of O_2_ for 0 or 2 hours with or without 0.2 mM H_2_O_2_ and 0.5 mM NAC pre-treatment.
(d) RT-qPCR analysis of BHLHE40 mRNA levels in MDA-MB-231 and MDA-MB-468 cells under 1% of O_2_ for 0 or 2 hours with or without 0.2 mM H_2_O_2_ and 0.5 mM NAC pre-treatment.
(e) Western blot analysis of BHLHE40 and HIF-1α proteins in MDA-MB-468 cells under 1% of O_2_ for 0 to 2 hours.
(f) Western blot analysis of BHLHE40, HIF-1α and BNIP3 proteins in MDA-MB-468 cells under 1% of O_2_ for 0, 2, 24, 48, or 72 hours.
(g) RT-qPCR analysis of HIF-1β mRNA levels in scramble control and HIF-1β knockdown subclones of MDA-MB-231 and MDA-MB-468 cells. #3, #4, different HIF-1β shRNA sequences.
(h) RT-qPCR analysis of BHLHE40 mRNA levels in scramble control and HIF-1β knockdown subclones of MDA-MB-468 cells under 1% of O_2_ for 0, 1, 2, or 24 hours.
(i) Western blot analysis of BHLHE40, HIF-1β, HIF-1α and BNIP3 proteins in scramble control and HIF-1β knockdown subclones of MDA-MB-468 cells under 1% of O_2_ for 0, 1, 2 or 24 hours.
(j) RT-qPCR analysis of BHLHE40 mRNA levels in MDA-MB-468 cells treated with or without 0.1 mM VH298.
(k) Western blot analysis of BHLHE40, HIF-1α, HIF-2α, HIF-1β and BNIP3 proteins in MDA-MB-468 cells treated with or without 0.1 mM VH298 under 1% of O_2_ for 0 or 2 hours.
(l) Graphic illustration of establishment of 4T1-BALB/c mouse model. n = 5, the number of mice.
(m) Body weight and tumor volume of 4T1-BALB/c mice.
(n) Raw image of 4T1-BALB/c mouse tumor samples on Day 26 after implantation.
(o) Pearson correlation analysis of BHLHE40 mRNA levels and mRNA-based hypoxia-signatures from literature (Buffa, Ragnum, West, Hu, and Seigneuric) in the TCGA_TNBC cohort.

hrs, hours. *p* values were determined by using one-way ANOVA (d, g, h; n = 3, mean ± SD), two-tailed unpaired Student’s t-tests (j; n = 3, mean ± SD) and Pearson correlation analysis (o; n = 187). **, *p* < 0.01; ***, *p* < 0.001; ****, *p* < 0.0001; ns, not significant.

Figure S4 Supplementary figures of Figure 5

(a) GO enrichment analysis of RNA-seq for DEGs between scramble control and BHLHE40 knockdown subclones of MDA-MB-231 cells under 1% of O_2_ for 24 hours.
(b) KEGG enrichment analysis of RNA-seq for DEGs between scramble control and BHLHE40 knockdown subclones of MDA-MB-231 cells under 1% of O_2_ for 24 hours.
(c) Gene set enrichment analysis (GSEA) of RNA-seq for DEGs between scramble control and BHLHE40 knockdown subclones of MDA-MB-231 cells under 1% of O_2_ for 24 hours. resp., response. NES, normalized enrichment score. All FDR < 0.05.

Figure S5 Supplementary figures of Figure 5

(a) Flow cytometry of intracellular ROS levels in scramble control and BHLHE40 knockdown subclones of MDA-MB-231 cells under 1% of O_2_ for 0 or 2 hours. DCF, 2',7'-dichlorofluorescein. A, area.
(b) Flow cytometry of intracellular ROS levels in scramble control and BHLHE40 knockdown subclones (shRNA #2) of MDA-MB-231 cells under 1% of O_2_ for 0 or 24 hours.
(c) RT-qPCR analysis of SVCT2, SOD2/3, PRDX1/2/5, GPX3/4, GLRX/GLRX2 and CAT mRNA levels in scramble control and BHLHE40 knockdown subclones of MDA-MB-231 cells under 1% of O_2_ for 0 or 24 hours.
(d) Flow cytometry of cell-surface xCT/SLC7A11 levels in scramble control and BHLHE40 knockdown subclones of MDA-MB-231 cells under 1% of O_2_ for 0 or 24 hours.
(e) Statistical analysis of cell-surface xCT/SLC7A11 flow cytometry assay.
(f) ChIP-qPCR analysis of BHLHE40 occupancy at the *GLRX* promoter in MDA-MB-231 cells under 1% of O_2_ for 0 or 24 hours. IgG served as a negative control.
(g) Western blot analysis of 4-HNE in scramble control and BHLHE40 knockdown subclones of MDA-MB-231 cells under 1% of O_2_ for 0 or 24 hours.

hrs, hours. p values were determined by using one-way ANOVA (a, b, c, e, f; n = 3, mean ± SD). *, p < 0.05; **, p < 0.01; ***, p < 0.001; ****, p < 0.0001; ns, not significant.

Figure S6 Supplementary figures of Figure 6

(a) RT-qPCR analysis of BHLHE40 mRNA levels in scramble control and BHLHE40 knockdown subclones of MDA-MB-468 cells. #2, #4, different BHLHE40 shRNA sequences.
(b) Western blot analysis of BHLHE40 proteins in scramble control and BHLHE40 knockdown subclones of MDA-MB-468 cells.
(c) CCK-8 assay of MDA-MB-231 and MDA-MB-468 cells 24, 48 or 72 hours after exposure to different doses of irradiation.
(d) Clonogenic assay of MDA-MB-231 and MDA-MB-468 cells 14 days after exposure to different doses of irradiation.
(e) Clonogenic assay of scramble control and BHLHE40 knockdown subclones (shRNA #2) of MDA-MB-231 cells under 1% of O_2_ for 0 or 24 hours, then 14 days after exposure to 2Gy doses of irradiation.
(f) Clonogenic assay of scramble control and BHLHE40 knockdown MDA-MB-231 cells reconstituted with wild-type (WT), C143A, or C342A BHLHE40, under 1% of O_2_ for 0 or 24 hours, then 14 days after exposure to 2Gy doses of irradiation.

*p* values were determined by using one-way ANOVA (a, d, e, f; n = 3, mean ± SD) and two-way ANOVA (c; n = 3, mean ± SD). *, *p* < 0.05; **, *p* < 0.01; ***, *p* < 0.001; ****, *p* < 0.0001.

Figure S7 Supplementary figures of Figure 7

(a) Body weight of MDA-MB-231-NOD/SCID mice. Data are presented as the mean ± SD (n=10 in shScramble/n=9 in shBHLHE40). IR, irradiation.

| Supporting Information, Table S1 | |
| --- | --- |
| Gene | Coefficient |
| TKTL1 | 0.447494 |
| ZFP36 | 0.282145 |
| PGF | 0.390734 |
| TGFBI | 0.119994 |
| CCN5 | 0.077186 |
| HAS1 | 0.077364 |
| SERPINE1 | 0.019131 |

Table S1 LASSO regression of seven hypoxia-prognosis related genes and their coefficients in the TCGA_TNBC cohort.

Supporting Information, Table S2

54 hypoxia-upregulated genes between hypoxia^high^ and hypoxia^low^ groups in the TCGA_TNBC cohort

CCN5, DUSP1, GPC1, CAVIN1, ANXA2, TPST2, HAS1, TGFBI, BGN, S100A4, SLC2A3, ETS1, PCK1, DCN, PDGFB, CAV1, PIM1, PLAUR, FOS, CCN2, CDKN1A, SRPX, SDC2, BHLHE40, SERPINE1, ADM, IL6, TGFB3, ATF3, PYGM, ANGPTL4, ZFP36, ACKR3, LOX, CA12, PFKFB3, COL5A1, HOXB9, JUN, HMOX1, P4HA2, SLC2A5, TKTL1, AKAP12, IER3, MT2A, PPP1R15A, TGM2, PGF, CAVIN3, CCN1, STBD1, KLF6, CITED2

Table S2 54 hypoxia-upregulated genes between hypoxia^high^ and hypoxia^low^ groups in the TCGA_TNBC cohort.

Supporting Information, Table S3

44 hypoxia-upregulated genes between 1% of O_2_ for 24 hours and 20% of O_2_ for 24 hours groups in TNBC cell lines

PPFIA4, SLC2A1, P4HA2, PGM1, NDRG1, TMEM45A, PFKFB3, VEGFA, ANGPTL4, VLDLR, RORA, BHLHE40, P4HA1, ADM, ALDOA, ERO1A, STC1, FAM162A, ANKZF1, FOS, BNIP3L, HK2, CCNG2, GAPDH, KDM3A, IGFBP3, CITED2, GYS1, LOX, LDHA, DDIT4, NFIL3, AK4, PDK1, GBE1, PLIN2, ALDOC, PGK1, GPI, STBD1, ENO2, EFNA3, MXI1, TPI1

Table S3 44 hypoxia-upregulated genes between 1% of O_2_ for 24 hours and 20% of O_2_ for 24 hours groups in TNBC cell lines.

| Supporting Information, Table S4 | | | | | | | | |
| --- | --- | --- | --- | --- | --- | --- | --- | --- |
| Exp. | No. | Sex | Age | Clinical diagnosis | HER-2 | ER | PR | FISH |
| IF | - | Female | 53 | Right breast cancer | ++ | - | - | - |
| WB | P1 | Female | 48 | Right breast cancer | ++ | - | - | - |
| WB | P2 | Female | 37 | Left breast cancer | ++ | - | - | - |
| WB | P3 | Female | 58 | Right breast cancer | + | - | - | - |
| WB | P4 | Female | 60 | Left breast cancer | + | - | - | - |
| WB | P5 | Female | 50 | Left breast cancer | + | - | - | - |
| WB | P6 | Female | 66 | Right breast cancer | + | - | - | - |

Table S4 All human samples information. Exp., the experiment applied of the human samples. No., the code of human samples.

| Supporting Information, Table S5 | | | | |  |
| --- | --- | --- | --- | --- | --- |
| Antibody | Brand name | Catalog | Dilution ratio | Be used in | |
| BHLHE40 (M) | Santa Cruz Biotechnology  (Texas, USA) | sc-101023 | 1: 50 | Fig. 2d | |
| TOM20 (R) | Proteintech Group | 11802-1-AP | 1: 50 | Fig. 2d | |
| HIF-1α (R) | Novus Biologicals  (Colorado, USA) | NB100-479 | 1: 100 | Fig. 4k | |
| BHLHE40 (R) | Proteintech Group | 17895-1-AP | 1: 50 | Fig. 2a-c, 4k-l | |
| BNIP3 (M) | Proteintech Group | 68091-1-Ig | 1: 500 | Fig. 4l | |

Table S5 All primary antibodies information used in immunoelectron microscopy, IF and IHC experiments. M, antibody source is mouse. R, antibody source is rabbit.

| Supporting Information, Table S6 | | | | |
| --- | --- | --- | --- | --- |
| Antibody | Brand name | Catalog | Dilution ratio | Be used in |
| IgG (R) | Proteintech Group | B900610 | 0.75[μg] antibody: 0.5[mg] protein | Fig. 3b (IP) |
| BHLHE40 (R) | Proteintech Group | 17895-1-AP | 0.75[μg] antibody: 0.5[mg] protein | Fig. 3b (IP) |
| BHLHE40 (M) | Santa Cruz Biotechnology | sc-101023 | 1: 500 | Fig. 3b (IB) |
| VDAC1 (R) | Proteintech Group | 10866-1-AP | 1: 500 | Fig. 3b (IB), 3f, S2f |
| BHLHE40 (R) | Proteintech Group | 17895-1-AP | 1: 500 | Fig. 3a, 3f-i, 4a-f, 4h, 4j, 5l, 6f, 7b, 7g, S1b, S2f, S3a-c, S3e-f, S3i, S3k, S6b |
| TUBULIN (R) | Proteintech Group | 11224-1-AP | 1: 2000 | Fig. 3g, 4a-b, 4h, 5e, 5l, 6f, S3a, S3i |
| LAMIN B1 (M) | Proteintech Group | 66095-1-Ig | 1: 20000 | Fig. 4a-b, S3a |
| TOM20 (R) | Proteintech Group | 11802-1-AP | 1:5000 | Fig. 3a, 4b |
| HIF-1α (R) | Cayman  (Michigan, USA) | 10009269 | 1: 2000 | Fig. 4c, 4e-f, 4h, 4j, 5l, S3c, S3e-f, S3i, S3k |
| HIF-1β (R) | Novus Biologicals | NB100-110 | 1: 2000 | Fig. 4c, 4h, 4j, S3c, S3i, S3k |
| BNIP3 (M) | Proteintech Group | 68091-1-Ig | 1: 5000 | Fig. 4f, 4h, 4j, 5l, S3f, S3i, S3k |
| HIF-2α (R) | BETHYL  (Texas, USA) | A700-003-T | 1: 1000 | Fig. 4h, 4j, S3k |
| SLC7A11 (R) | Proteintech Group | 26864-1-AP | 1: 500 | Fig. 5e |
| ACTIN (M) | Proteintech Group | 66009-1-Ig | 1: 2000 | Fig. 3h-i, 4a, 4c-f, 4j, 7b, 7g, S1b, S3a-c, S3e-f, S3k, S5g, S6b |
| SLC7A11 (R) | Alomone Labs | ANT-111-PE | 5 [μg]/10^6^ cells | Fig. S5d |
| 4-HNE (R) | abcam | ab46545 | 1:1000 | Fig. S5g |
| TIM23 (R) | Proteintech Group | 11123-1-AP | 1:1000 | Fig. 3a |
| TFAM (R) | Proteintech Group | 22586-1-AP | 1:5000 | Fig. 3a |

Table S6 All primary antibodies information used in IP, WB and flow cytometry experiments. M, antibody source is mouse. R, antibody source is rabbit.

| Supporting Information, Table S7 | |
| --- | --- |
| Primer | Sequence (5’ to 3’) |
| HIF-1β (F) | CTGCCAACCCCGAAATGACAT |
| HIF-1β (R) | CGCCGCTTAATAGCCCTCTG |
| BHLHE40 (F) | AGCAGTGGTTCTTGAACTTACC |
| BHLHE40 (R) | ACAAGCTGCGAAGACTTCAGG |
| 18S rRNA (F) | CGGCGACGACCCATTCGAAC |
| 18S rRNA (R) | GAATCGAACCCTGATTCCCCGTC |
| SVCT2 (F) | CTTCACTCTTCCGGTGGTGAT |
| SVCT2 (R) | TTTCCGTAGTGTAGATCGCCA |
| SOD2 (F) | GCTCCGGTTTTGGGGTATCTG |
| SOD2 (R) | GCGTTGATGTGAGGTTCCAG |
| PRDX5 (F) | GCAAGACGGTGCAGTGAAG |
| PRDX5 (R) | ATGGCATCTCCCACCTTGATT |
| GPX4 (F) | GAGGCAAGACCGAAGTAAACTAC |
| GPX4 (R) | CCGAACTGGTTACACGGGAA |
| PRDX2 (F) | GAAGCTGTCGGACTACAAAGG |
| PRDX2 (R) | TCGGTGGGGCACACAAAAG |
| GLRX (F) | CCCATCAAACAAGGGCTTCTG |
| GLRX (R) | CTGCATCCGCCTATACAATCTT |
| SOD3 (F) | ATGCTGGCGCTACTGTGTTC |
| SOD3 (R) | CTCCGCCGAGTCAGAGTTG |
| GLRX2 (F) | TCTTTGGAGAATTTAGCGACGG |
| GLRX2 (R) | CTGGTTTCCATATTCAAGCAGGT |
| PRDX1 (F) | CCACGGAGATCATTGCTTTCA |
| PRDX1 (R) | AGGTGTATTGACCCATGCTAGAT |
| CAT (F) | TGGAGCTGGTAACCCAGTAGG |
| CAT (R) | CCTTTGCCTTGGAGTATTTGGTA |
| GPX3 (F) | AGAGCCGGGGACAAGAGAA |
| GPX3 (R) | ATTTGCCAGCATACTGCTTGA |
| SLC7A11 (ChIP-qPCR-F) | CAGCAGCAGTGGTGGAACGA |
| SLC7A11 (ChIP- qPCR-R) | AAACTCAAAGGTGTGCTTTT |
| GLRX (ChIP- qPCR-F) | AAAGAGAGGAGGCTCAGACTC |
| GLRX (ChIP- qPCR-R) | AGCCAGATTCTGAGTCACGGA |

Table S7 All primers information used in RT-qPCR and ChIP- qPCR experiments. F, forward primer. R, reverse primer.

| Supporting Information, Table S8 | |
| --- | --- |
| shRNA | Sequence (5’ to 3’) |
| HIF-1β (#3) | TGTTGGCTACCAGCCACAGGAACTCTTAG |
| HIF-1β (#4) | TCAGATGTCTAACGATAAGGAGCGGTTTG |
| BHLHE40 (#2) | GGTCAAGAGATGTTCTGCTCAGGTTTCCA |
| BHLHE40 (#4) | CTGTCTTGCTCCAAGCTCTGAAGCCAATC |

Table S8 All shRNA information used in lentivirus transduction experiments.

| Supporting Information, Table S9 | | | | | | | | | | | | |
| --- | --- | --- | --- | --- | --- | --- | --- | --- | --- | --- | --- | --- |
| TPMs | 1% O_2_ 0 hrs | | | | | | 1% O_2_ 24 hrs | | | | | |
|  | shScramble | | | shBHLHE40 (#4) | | | shScramble | | | shBHLHE40 (#4) | | |
|  | Rep1 | Rep2 | Rep3 | Rep1 | Rep2 | Rep3 | Rep1 | Rep2 | Rep3 | Rep1 | Rep2 | Rep3 |
| SVCT2 | 17.94 | 13.65 | 15.21 | 15.74 | 16.48 | 17.33 | 12.17 | 17.09 | 17.8 | 14.17 | 13.72 | 15.92 |
| SOD2 | 102.13 | 77.91 | 80.34 | 77.62 | 79.8 | 91.82 | 79.66 | 96.67 | 104.96 | 88.09 | 86.91 | 92.64 |
| PRDX5 | 235.51 | 239.57 | 240.17 | 222.07 | 226.34 | 210.58 | 288.99 | 273.96 | 276.76 | 253.88 | 244.52 | 261.39 |
| GPX4 | 575.06 | 652.84 | 599.31 | 563.85 | 562.16 | 567.74 | 619.2 | 632.19 | 604.86 | 623.53 | 604.1 | 599.53 |
| PRDX2 | 454.6 | 481.24 | 431.09 | 417.97 | 408.46 | 419.92 | 482.95 | 453.1 | 442.01 | 408.08 | 448.42 | 413.74 |
| GLRX | 155.94 | 162.2 | 158.28 | 74.65 | 82.01 | 77.13 | 223.01 | 222.15 | 229.81 | 92.69 | 91.81 | 87.77 |
| SOD3 | 22.96 | 19.91 | 17.75 | 3.29 | 2.66 | 3.44 | 23.04 | 22.48 | 22.43 | 4.33 | 3.33 | 3.71 |
| GLRX2 | 71.63 | 84.7 | 81.02 | 66.87 | 64.29 | 66.4 | 71.2 | 66.52 | 73.3 | 59.83 | 61.16 | 60.6 |
| PRDX1 | 2164.62 | 2206.31 | 2270.76 | 1953.18 | 1909.76 | 1899.06 | 2158.12 | 1958.14 | 1964.23 | 1854.28 | 1807.2 | 1796.93 |
| CAT | 67.42 | 65.81 | 62.79 | 57.33 | 59.27 | 55.64 | 65.39 | 64.33 | 66.21 | 47.37 | 49.56 | 48.87 |
| GPX3 | 187.4 | 198.62 | 172.3 | 136.91 | 136.11 | 136.19 | 172.67 | 192.44 | 179.74 | 123.56 | 138.45 | 125.68 |

Table S9 Supporting information of Figure 6a.

Tumor weight data of Fig. 7c

| Tumor weight (g) | shScramble | shScramble-IR | shBHLHE40 | shBHLHE40-IR |
| --- | --- | --- | --- | --- |
|  | 1.02 | 0.44 | 1.25 | 0.17 |
|  | 1.01 | 0.37 | 1.19 | 0.19 |
|  | 0.98 | 0.35 | 1.27 | 0.16 |
|  | 1.00 | 0.38 | 1.26 | 0.15 |
|  | 0.94 | 0.31 | 1.24 | 0.20 |
|  | 0.99 | 0.34 | 1.20 | 0.13 |
|  | 1.11 | 0.42 | 1.23 | 0.16 |
|  | 1.09 | 0.38 | 1.19 | 0.22 |
|  | 1.02 | 0.39 | 1.14 | 0.14 |
|  | 0.92 | 0.35 |  |  |

Tumor weight data of MDA-MB-231-NOD/SCID mice in Fig. 7c. Data are presented as the mean ± SD (n=10 in shScramble/n=9 in shBHLHE40). *p* values were determined by using one-way ANOVA. ****, *p* < 0.0001. Scrambled control or BHLHE40 knockdown MDA-MB-231 cells were injected into the mammary fat pads (MFPs) of NOD/SCID mice. Tumors were locally irradiated with 20 Gy X-rays once they reached 100 mm^3^, and mice were euthanized 21 days later. Two shBHLHE40-injected mice died before tumor engraftment and were excluded prior to irradiation, leaving n = 9 for both shBHLHE40 and shBHLHE40-IR groups; all other groups had n = 10.

Tumor weight data of Fig. S3n

| Tumor weight (g) | F7 | F8 | F9 | F12 | F13 |
| --- | --- | --- | --- | --- | --- |
|  | 0.84 | 0.98 | 1.12 | 0.14 | 0.97 |

Tumor weight data of 4T1-BALB/C mice in Fig. S3n.
