# Supplementary figures and images for: Intratumoral Hypoxia Triggers Mitochondrial BHLHE40 ROS Sensing Pathway to Promote Radioresistance in Triple‐Negative Breast Cancer
Source: Adv Sci (Weinh). 2026 Jul 27:e76864. Online ahead of print. doi: 10.1002/advs.76864 (PMC13403735; doi:10.1002/advs.76864)

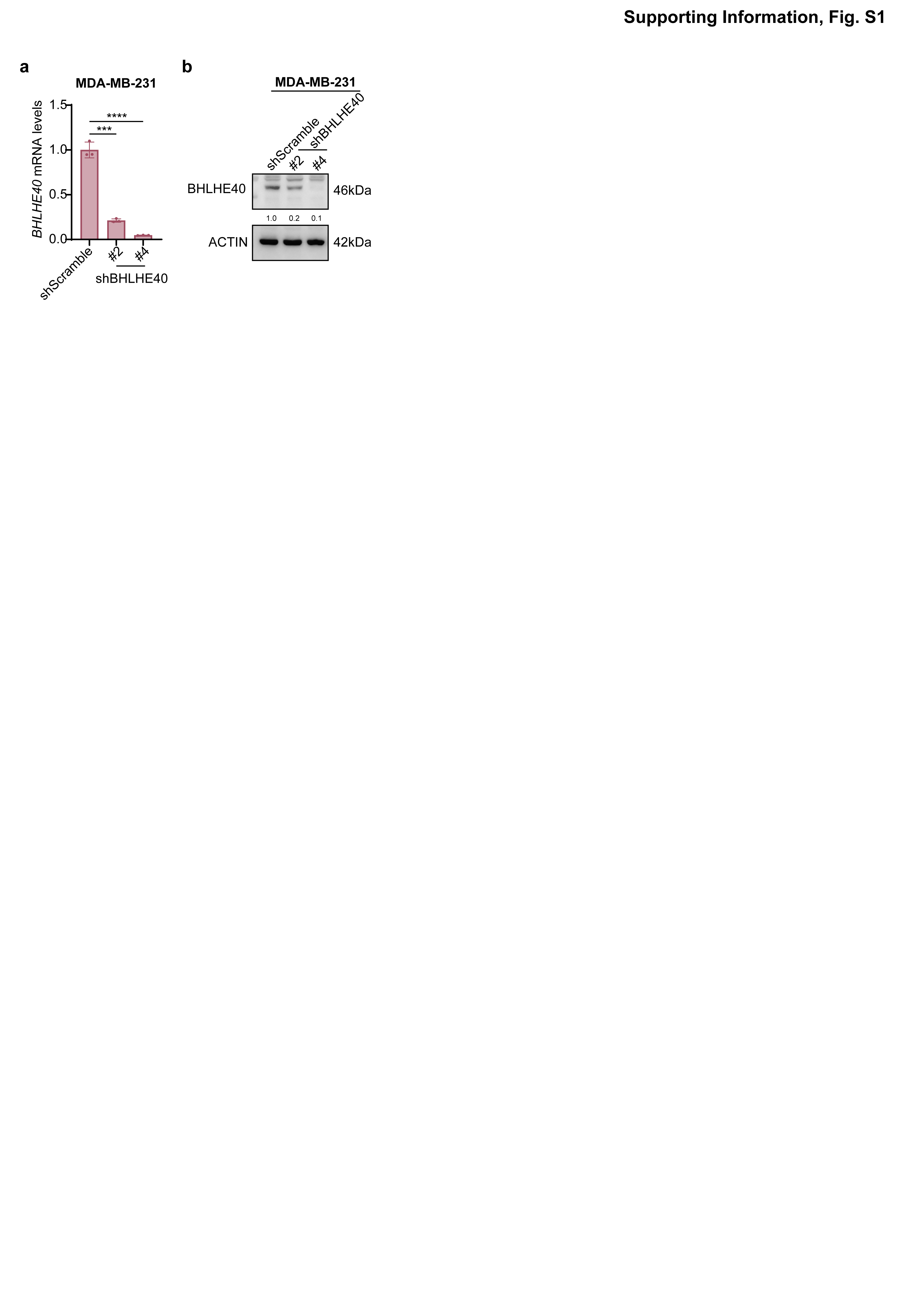

Supplement: Supplementary file 2 — Supporting File 2: advs76864‐sup‐0002‐FigureS1‐S7.zip. [file ADVS-9999-e76864-s002.zip › Supporting Information, Figure S1.jpg]

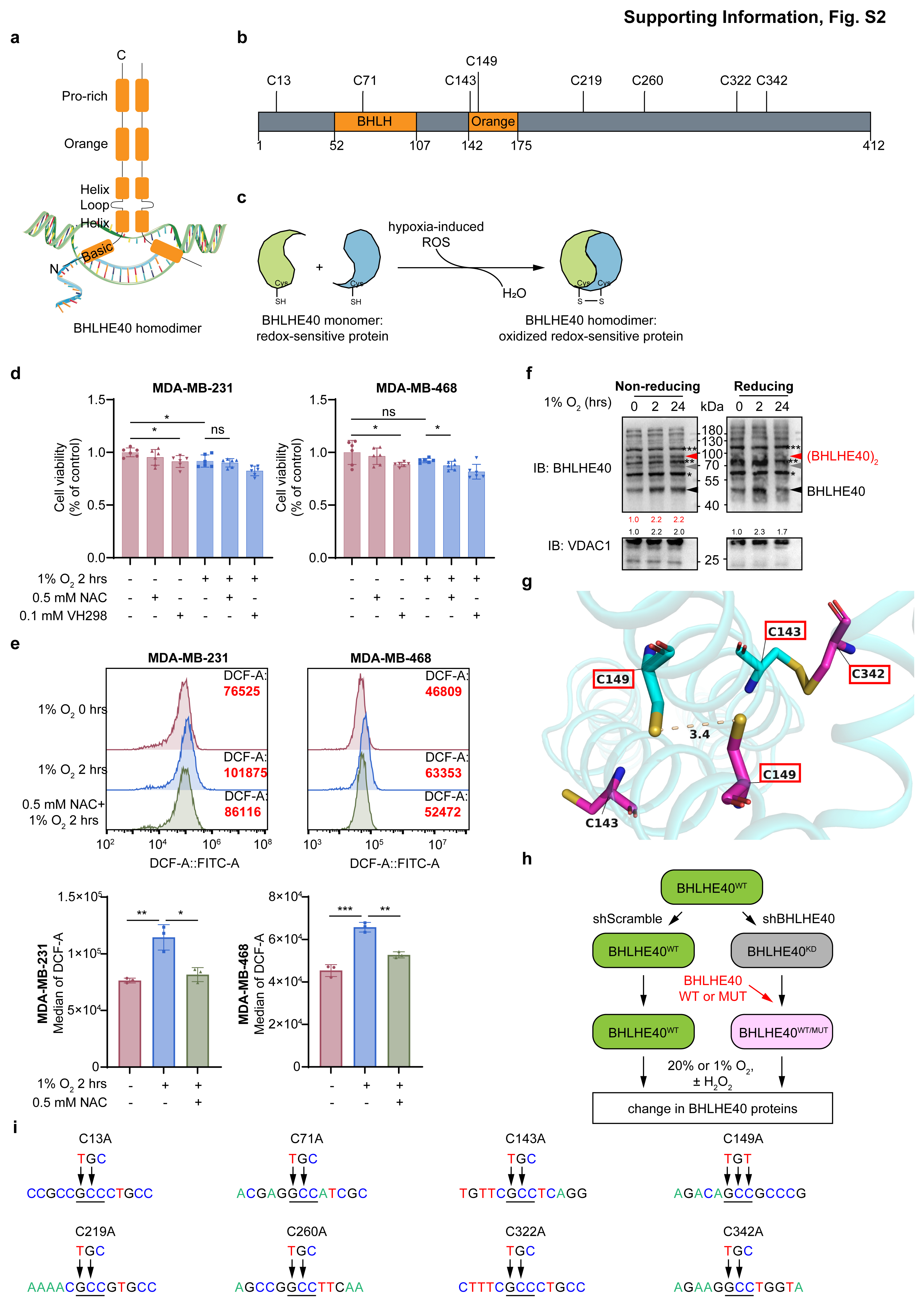

Supplement: Supplementary file 2 — Supporting File 2: advs76864‐sup‐0002‐FigureS1‐S7.zip. [file ADVS-9999-e76864-s002.zip › Supporting Information, Figure S2.jpg]

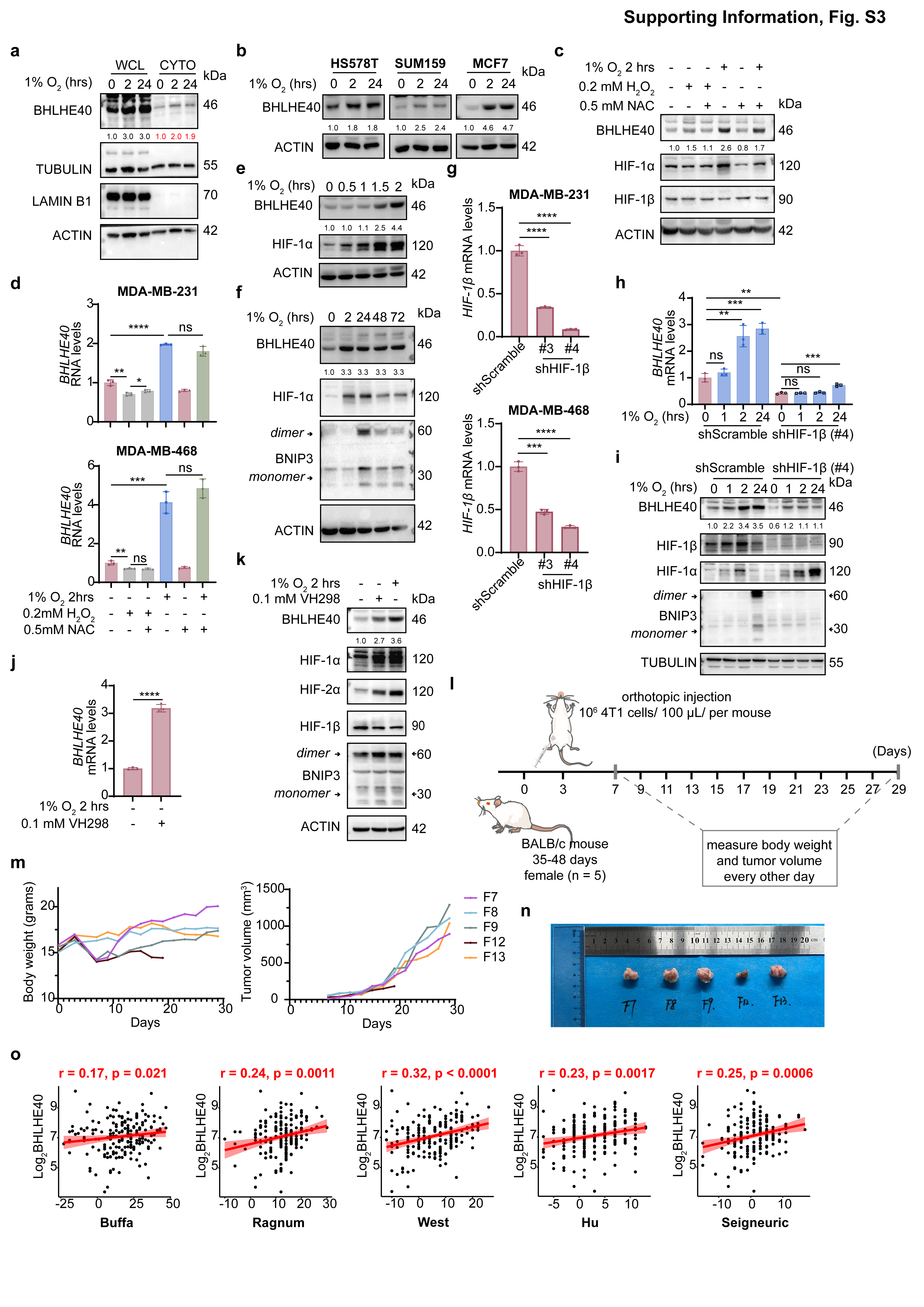

Supplement: Supplementary file 2 — Supporting File 2: advs76864‐sup‐0002‐FigureS1‐S7.zip. [file ADVS-9999-e76864-s002.zip › Supporting Information, Figure S3.jpg]

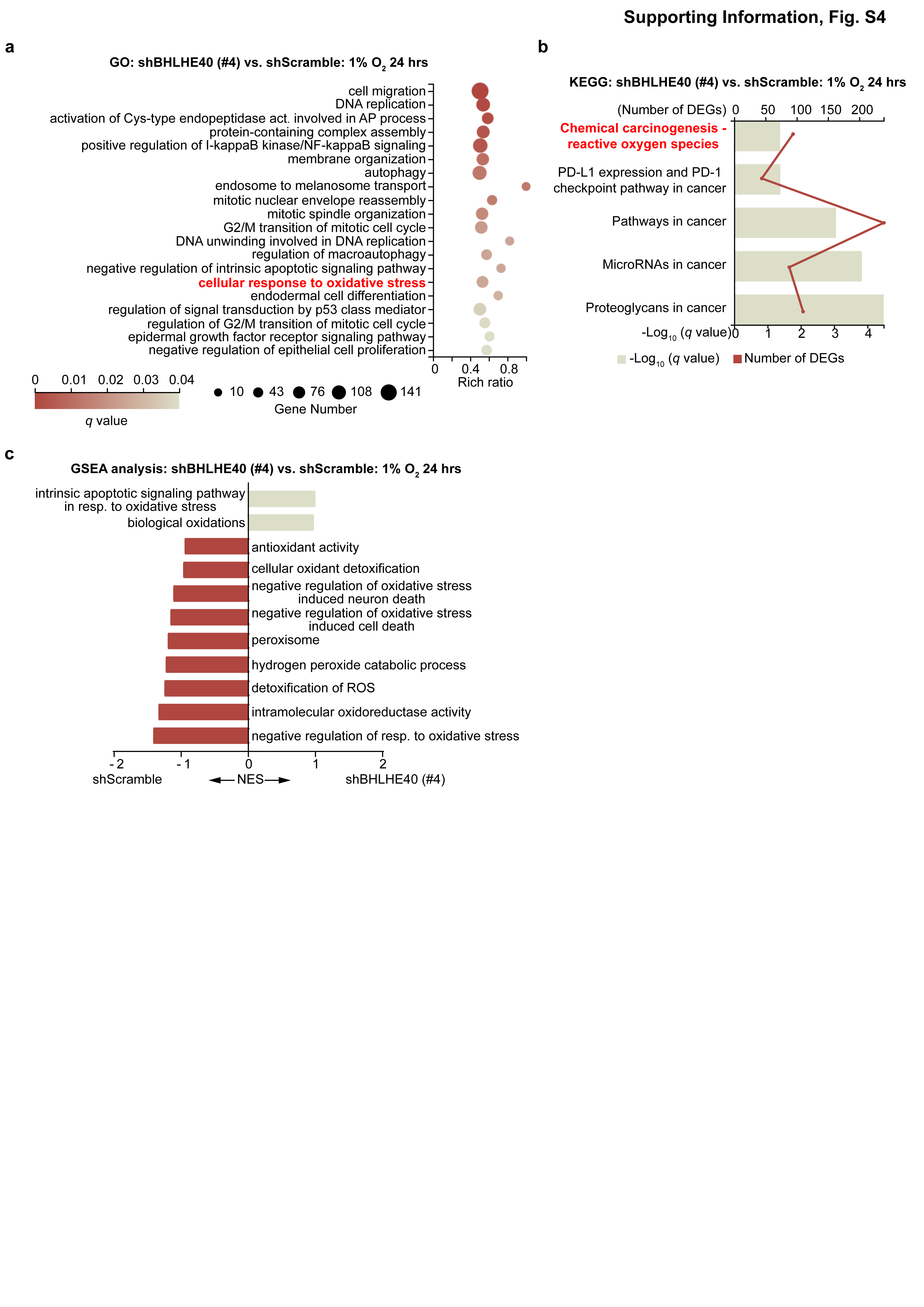

Supplement: Supplementary file 2 — Supporting File 2: advs76864‐sup‐0002‐FigureS1‐S7.zip. [file ADVS-9999-e76864-s002.zip › Supporting Information, Figure S4.jpg]

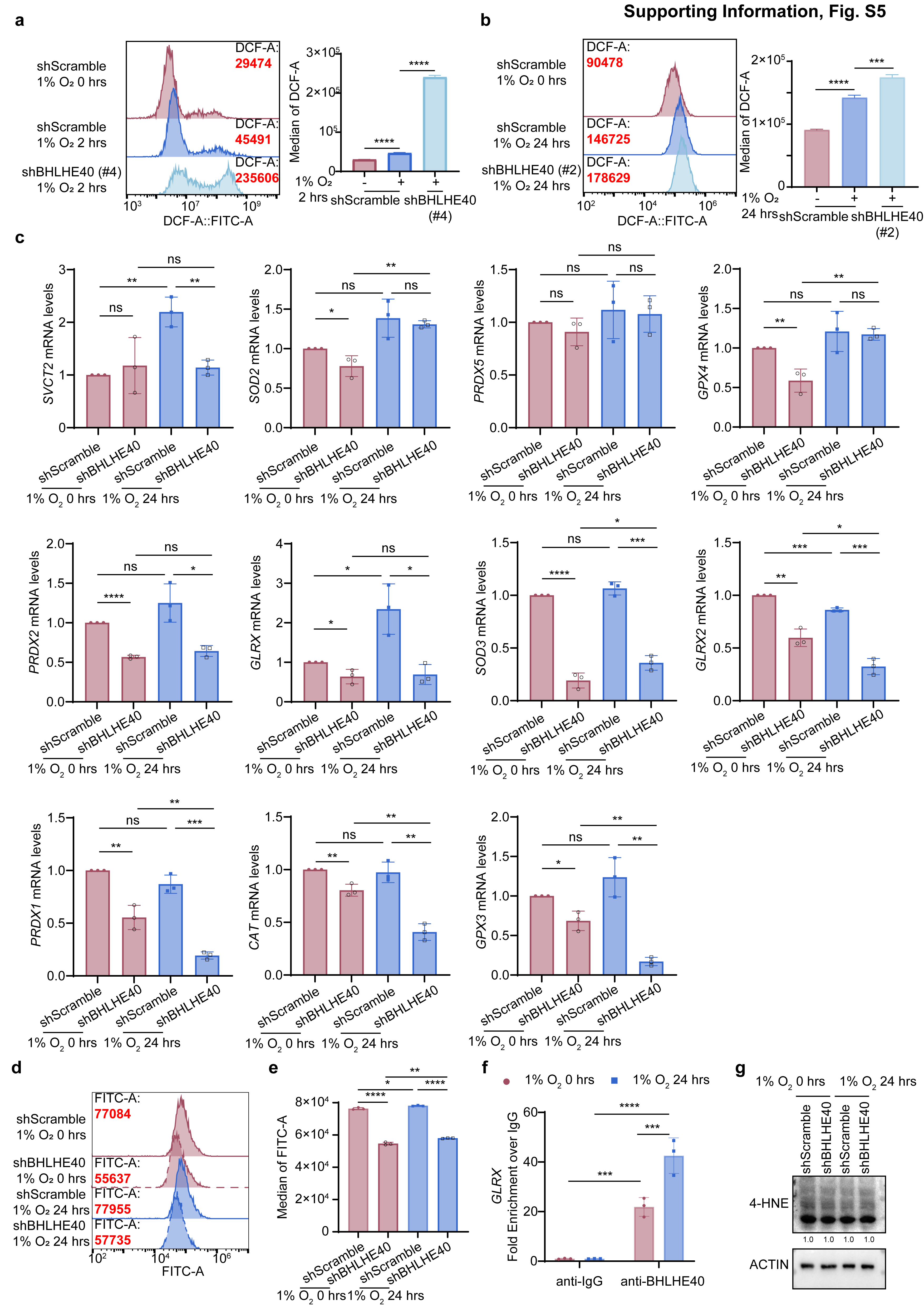

Supplement: Supplementary file 2 — Supporting File 2: advs76864‐sup‐0002‐FigureS1‐S7.zip. [file ADVS-9999-e76864-s002.zip › Supporting Information, Figure S5.jpg]

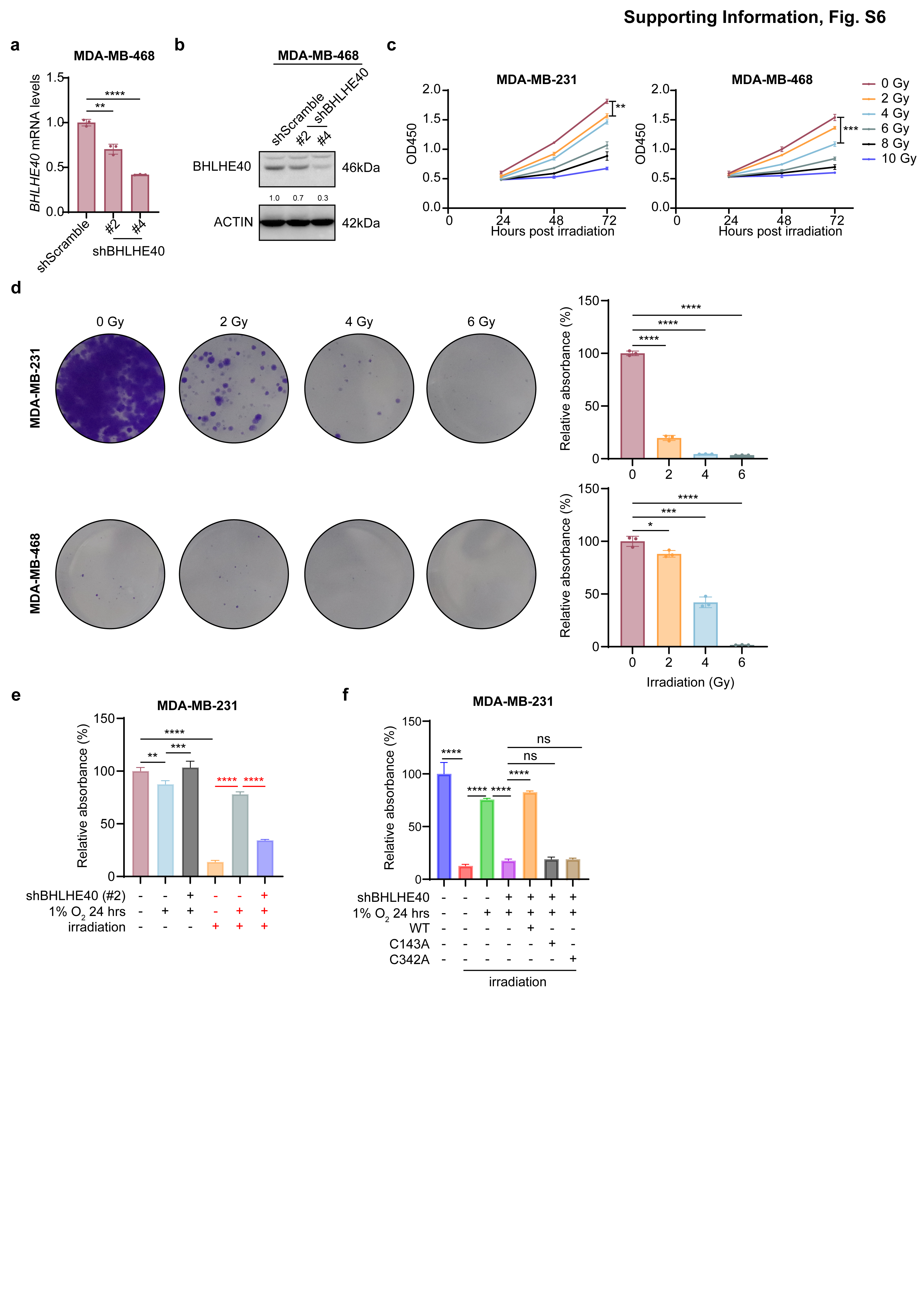

Supplement: Supplementary file 2 — Supporting File 2: advs76864‐sup‐0002‐FigureS1‐S7.zip. [file ADVS-9999-e76864-s002.zip › Supporting Information, Figure S6.jpg]

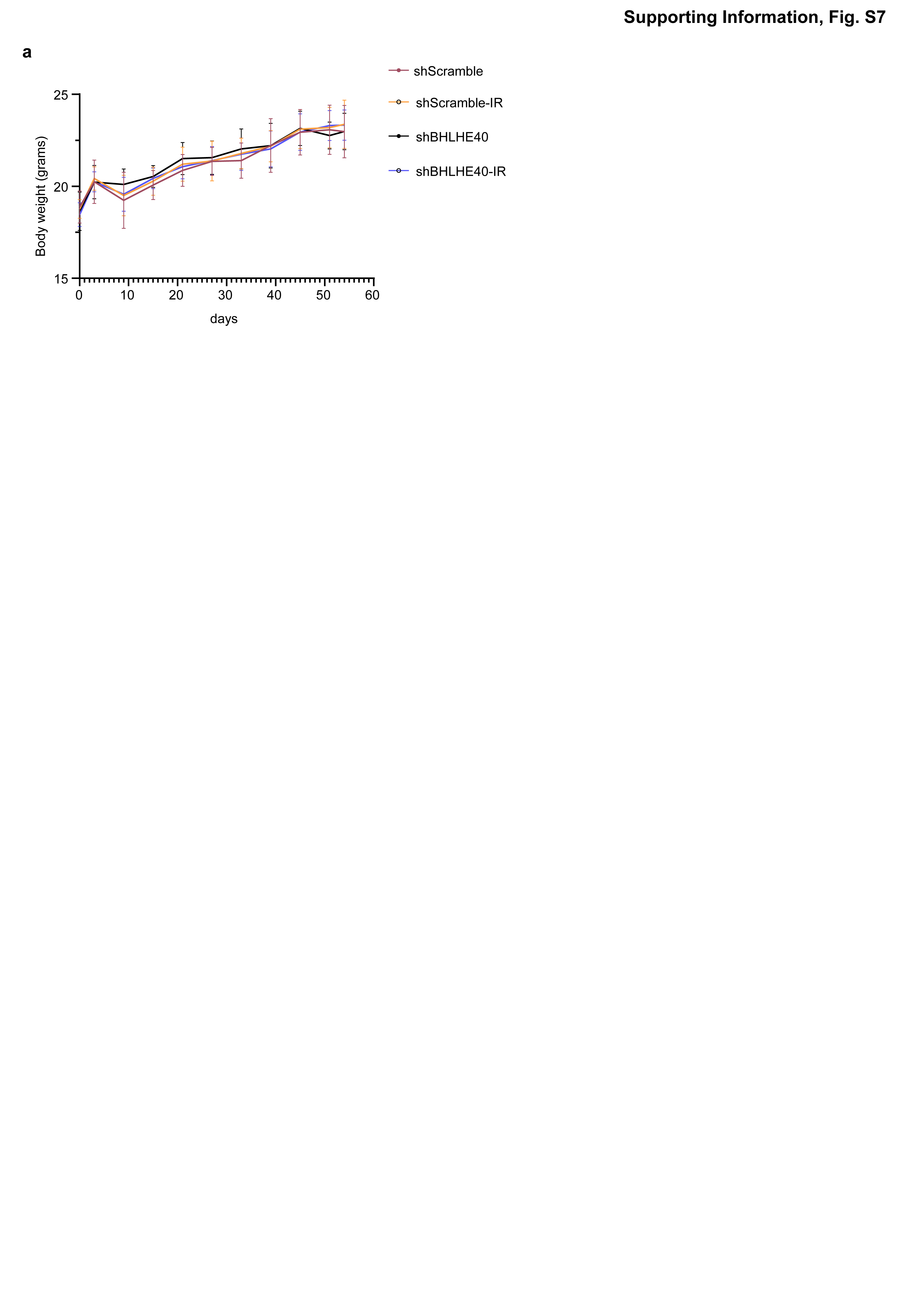

Supplement: Supplementary file 2 — Supporting File 2: advs76864‐sup‐0002‐FigureS1‐S7.zip. [file ADVS-9999-e76864-s002.zip › Supporting Information, Figure S7.jpg]
